# New species of *Daidalotarsonemus* and *Excelsotarsonemus* (Acari, Tarsonemidae) from the Brazilian rainforest

**DOI:** 10.3897/zookeys.475.8827

**Published:** 2015-01-22

**Authors:** José Marcos Rezende, Antonio Carlos Lofego, Ronald Ochoa, Gary Bauchan

**Affiliations:** 1 Programa de Pós-Graduação em Biologia Animal, São Paulo State University, São José do Rio Preto, SP 15054-000, Brazil; 2 Departamento de Zoologia e Botânica, São Paulo State University, São José do Rio Preto, SP 15054-000, Brazil; 3 Systematic Entomology Laboratory, United States Department of Agriculture, Agricultural Research Service, Beltsville, MD 20705, USA; 4 Electron and Confocal Microscopy Unit, United States Department of Agriculture, Agricultural Research Service, Beltsville, MD 20705, USA

**Keywords:** Atlantic Forest, canopy, faunistics, LT-SEM, systematics, Tarsonemoidea, Trombidiformes

## Abstract

Three new species of Tarsonemidae, *Daidalotarsonemus oliveirai* Rezende, Lofego & Ochoa, **sp. n.**, *Excelsotarsonemus caravelis* Rezende, Lofego & Ochoa, **sp. n.** and *Excelsotarsonemus tupi* Rezende, Lofego & Ochoa, **sp. n.** are described and illustrated. Measurements for these species are provided, as well as drawings, phase contrast (PC), differential interference contrast (DIC) and low temperature scanning electron microscopy (LT-SEM) micrographs. Some characters, which have not been used or clearly understood, are described herein. Biological, ecological and agricultural aspects about the role of these species in the rainforest and its surrounding environment are briefly discussed.

## Introduction

Currently, *Daidalotarsonemus* De Leon (Acari, Prostigmata, Tarsonemidae) consists of 26 described species (Lin and Zhang 2002, Lofego et al. 2005, Sousa et al. 2014). It is one of the few genera of Tarsonemidae which have been documented on all continents, except Antarctica (Lindquist 1986, Lin and Zhang 2002). The known geographical distribution of *Excelsotarsonemus* Ochoa & Naskręcki is much more restricted, previously recorded only from Costa Rica. The two genera are closely related and considered to be sister genera (Ochoa et al. 1995, Ochoa and OConnor 1998). Both are considered to be plant inhabiting taxa, apparently with a preference for plants located in humid places, where there is an abundance of fungi, bacteria and lichens. In Brazil, the Amazon and Atlantic Rainforests are biomes which fit these requirements, because of their high temperature and rainfall index.

In recent years, significant advances in microscopy have expanded our knowledge of the morphological characters of organisms, which has led to a better understanding of the taxonomy and ecology of species (Fisher and Dowling 2010). One of most effective techniques that has been integrated to study mite morphology and biology is Low Temperature Scanning Electron Microscopy (LT-SEM), in which a sample is instantly frozen with liquid nitrogen, making a frozen snap-shot of the specimen as it occurs in nature available for microscopic study (Bolton et al. 2014). This procedure is critical for understanding not only external morphology, but also ecological and behavior characteristics, not accessible using light microscopy.

The objective here is to describe new species of *Daidalotarsonemus* and *Excelsotarsonemus* found in a rainforest in Brazil using phase contrast (PC), differential interference contrast (DIC) light microscopy and LT-SEM microscopy techniques. The LT-SEM study led to a better understanding of the morphology of these species and their respective genera and is discussed herein.

## Material and methods

Several leaves of *Annona muricata* L. (Annonaceae), *Theobroma cacao* L. (Malvaceae) and *Spondias purpurea* L. (Anacardiaceae) were collected in and the area surrounding a section of the rainforest near Santa Cruz State University campus (UESC), 14°47'45"S; 39°10'18"W, Ilhéus, Bahia State, Brazil. The region is characterized by having high relative humidity (75–90%) and high precipitation (100–330 mm/month) indexes throughout the year. Mites collected in the study were prepared and analysed using three different microscopy techniques: phase contrast (PC), differential interference contrast (DIC) and low temperature scanning electron microscopy (LT-SEM). The terminology used herein follows that of Lindquist (1986), except fot the gnathosomal setae *dgs* and *vgs* (Magowski and Di Palma 2000). For each structure, all the measurements are provided in micrometers (µm), followed by the range of all specimens measured in parentheses, including the holotype. The following abbreviations are used for institutions where the types were deposited: Acari Collection of the Departamento de Zoologia e Botânica (DZSJRP), São Paulo State University, São José do Rio Preto, São Paulo, Brazil; United States National Museum of Natural History (USNM), Smithsonian Institution, housed in Beltsville, Maryland 20705, USA.

Specimens were prepared and observed with an LT-SEM using the same techniques as described in Bolton et al. (2014). Briefly, live specimens were secured to 15 cm × 30 cm copper plates using ultra smooth, round (12 mm diameter), carbon adhesive tabs (Electron Microscopy Sciences, Inc., Hatfield, PA). The specimens were frozen in a Styrofoam box, by placing the plates on the surface of a pre-cooled (-196 °C) brass bar whose lower half was submerged in liquid nitrogen (LN_2_). After 20–30 seconds, the holders containing the frozen samples were transferred the Quorum PP2000 cryo-prep chamber (Quorum Technologies, East Sussex, UK) attached to an S-4700 field emission scanning electron microscope (Hitachi High Technologies America, Inc., Dallas, TX). The specimens were etched inside the cryotransfer system to remove any surface contamination (condensed water vapour) by raising the temperature of the stage to -90 °C for 10–15 minutes. Following etching, the temperature inside the chamber was lowered below -130 °C, and the specimens were coated with a 10 nm layer of platinum using a magnetron sputter head equipped with a platinum target. The specimens were transferred to a pre-cooled (-130 °C) cryostage in the SEM for observation. An accelerating voltage of 5 kV was used to view the specimens. Images were captured using a 4pi Analysis System (Durham, NC). For the PC and DIC micrographs, it was used a Zeiss Axioscope™ microscope with differential interference contrast (DIC) 100× Plan Apochromatic objective with a NA 1.4. For the drawings, it was used a Leica^®^ DM 2500 microscope with a drawing tube attached. Images were sized and placed together to produce a single illustrative plate using the software Adobe® Photoshop CS 5.0 and Adobe® Illustrator CS 5.0.

## Results

### Daidalotarsonemus oliveirai

Taxon classificationAnimaliaTrombidiformesTarsonemidae

Rezende, Lofego & Ochoa sp. n.

http://zoobank.org/4929C981-2DE8-4145-9C2F-6FBF54B92F67

Figs 1 , 2 , 3 , 4 , 5 , 6 , 7 , 8 , 9 , 10 , 11 , 12 , 13 , 14

#### Diagnosis.

Females of the new species are most similar to those of *Daidalotarsonemus jamesbakeri* Smiley (1969) and *Daidalotarsonemus folisetae* Lofego & Ochoa (Lofego et al. 2005), because of the irregular ornamentation pattern on the prodorsum and the similar shape of the setae *e.* However, *Daidalotarsonemus oliveirai* sp. n. has the tergite C with a W-shaped reticulate pattern in central area and longitudinal, wavy interrupted ridges laterally, whereas in *Daidalotarsonemus jamesbakeri* and *Daidalotarsonemus folisetae* the reticulation is uniform on all tergites, with longitudinal continuous ridges. The shape of setae *e* is also different among the three species, being cordate in *Daidalotarsonemus oliveirai*, acicular in *Daidalotarsonemus jamesbakeri*, and phylliform in *Daidalotarsonemus folisetae*. Males are similar to *Daidalotarsonemus deleoni* Smiley (1967), by the shape and length of almost all dorsal setae, except the setae *sc1*. In *Daidalotarsonemus oliveirai*, the relative length of the setae *sc1*/*sc2* is 1:0.6, whereas in *Daidalotarsonemus deleoni* is 1:0.3.

#### Adult female

(6 specimens measured). Gnathosoma (Figs 3 and 6): partially covered by the prodorsum. Subtriangular in ventral view, length 24 (23–26), maximum width 21 (19–23); dorsal apodeme distinct. Setae *dgs* 9 (8–10) and *vgs* 6 (6) smooth; palps moderately long 9 (8–11), with two small subterminal setae and terminal projections. Pharynx fusiform, 19 (18–23) long and 6 (5–7) wide at maximum width. Gnathosoma, idiosoma and legs covered with tiny dimples, each around 0.3 (0.2–0.5) in diameter.

**Figure 1.**
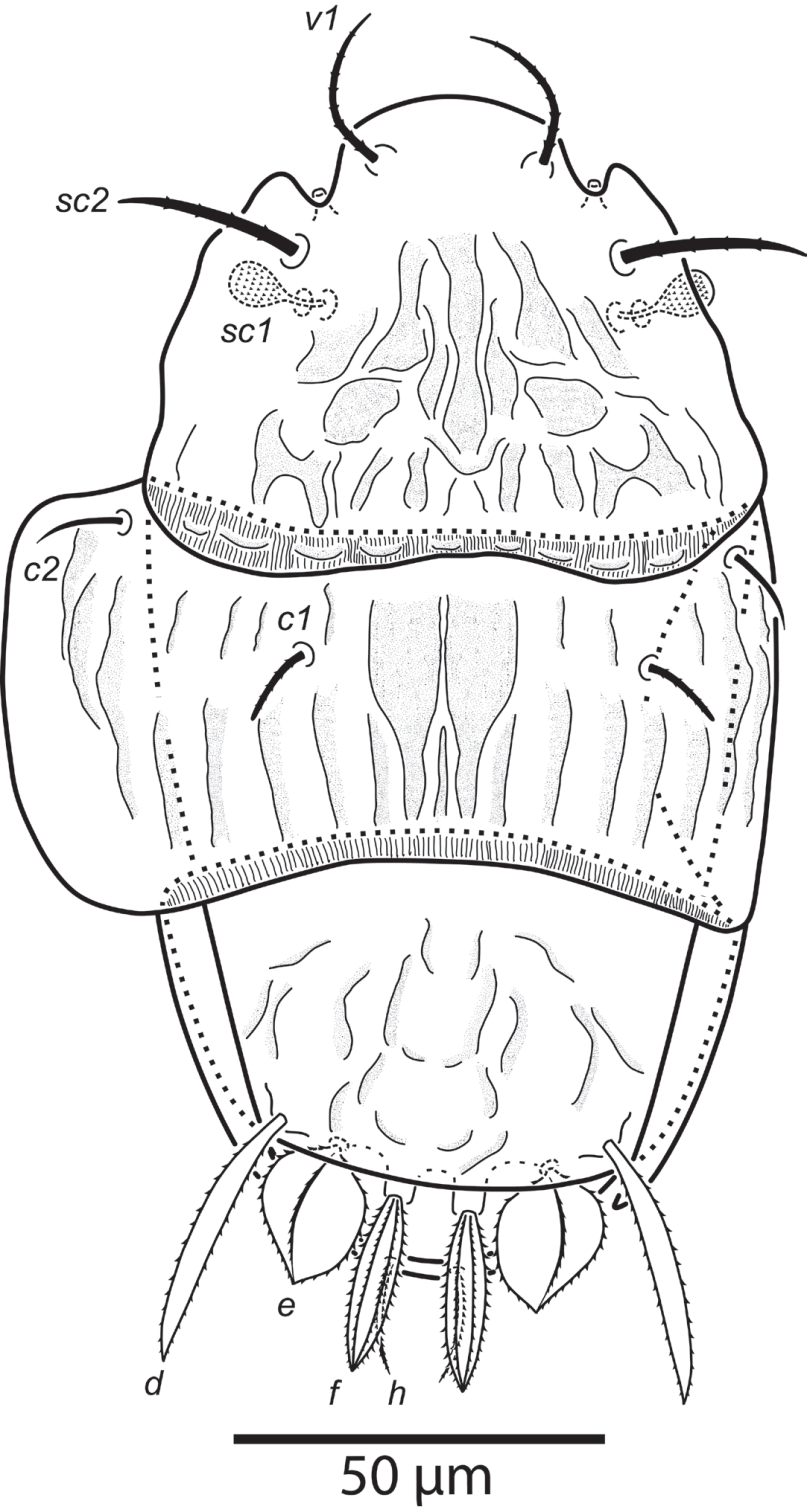
*Daidalotarsonemus oliveirai* sp. n. (female). Dorsal surface.

**Figure 2.**
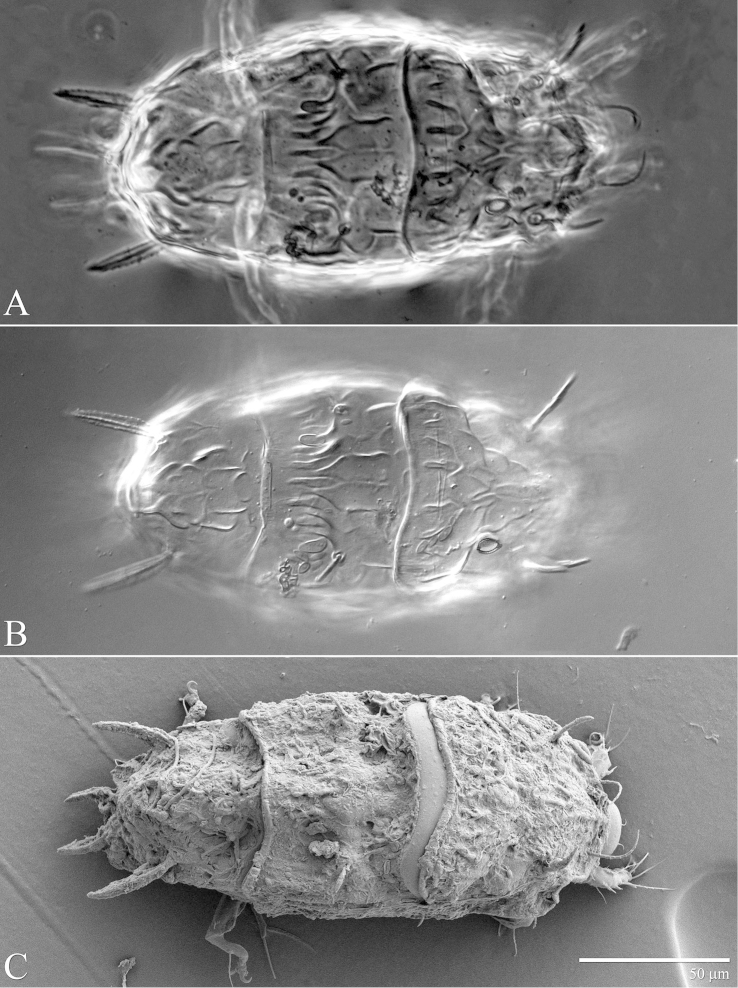
*Daidalotarsonemus oliveirai* sp. n. (female). Dorsal micrographs. **A** phase contrast **B** differential interference contrast **C** low temperature scanning electron microscopy.

Idiosoma – dorsum (Figs 1–2): length 179 (170–188), width at level of *c1* 82 (75–90); prodorsal shield with irregular ornamentation covers the gnathosoma. Entire dorsum covered with cerotegument (Fig. 2C). Stigma located near lateral notch of prodorsal shield, which is equidistant to the *v1* and *sc2* setal bases. Tergite C with a W-shaped reticulate pattern in central area and longitudinal, wavy uninterupted ridges laterally; tergite D ornamented with regular sculpturing. Lengths of the setae: *v1* 23 (22–25), *sc1* 11 (10–12) (Fig. 7C), *sc2* 28, *c1* 11 (10–12), *c2* 11 (10–12), *d* 33 (31–35), *e* 15 (15–16), *f* 24 (23–25) and *h* 24 (24–25). Maximum width of expanded setae: *d* 6 (5–7), *e* 15 (14–16) and *f* 7 (7–8). All dorsal setae serrate, except for *c2* smooth. Bothridial setae *sc1* capitate, with tiny spines. Setae *v1*, *sc2*, *c1*, *c2* and *h* setiform; setae *d*, *e* and *f* inserted on tubercles (Fig. 7D-E). Setae *d* linear and *e* cordate, both with a central serrate vein; *f* lanceolate, with two serrate veins. Distances between dorsal setae: *v1*–*v1* 25 (24–27), *sc2*–*sc2* 46 (44–48), *v1*–*sc2* 23 (23–24), *c1*–*c1* 50 (49–53), *c2*–*c2* 82 (76–88), *c1*–*c2* 27, *d*–*d* 44 (40–48), *f*–*f* 10, *e*–*f* 12 (11–13) and *h*–*h* 16 (14–17). Seta *sc2* inserted anteriorly to *sc1*. Dorsal cupules not easily seen.

**Figure 3.**
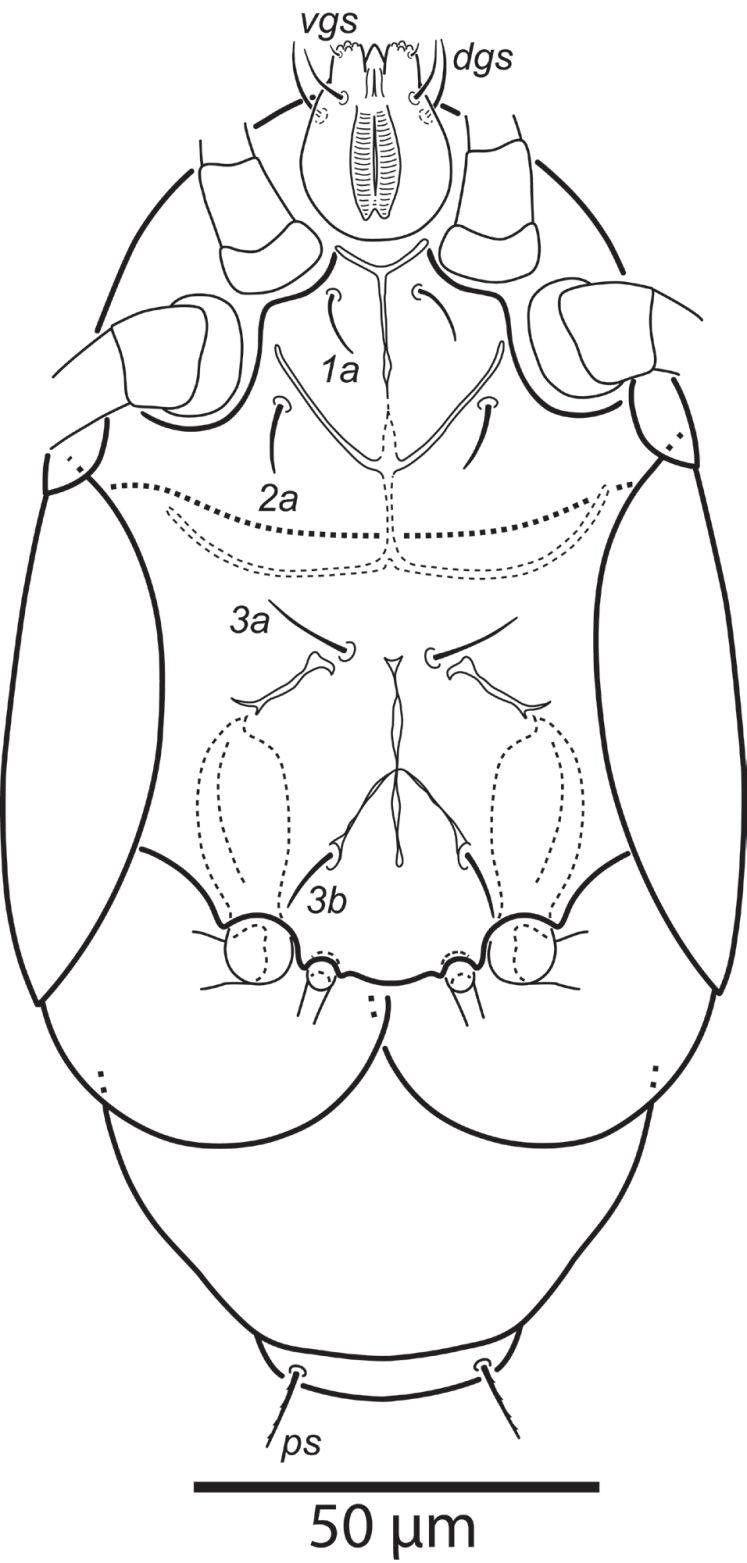
*Daidalotarsonemus oliveirai* sp. n. (female). Ventral surface.

**Figure 4.**
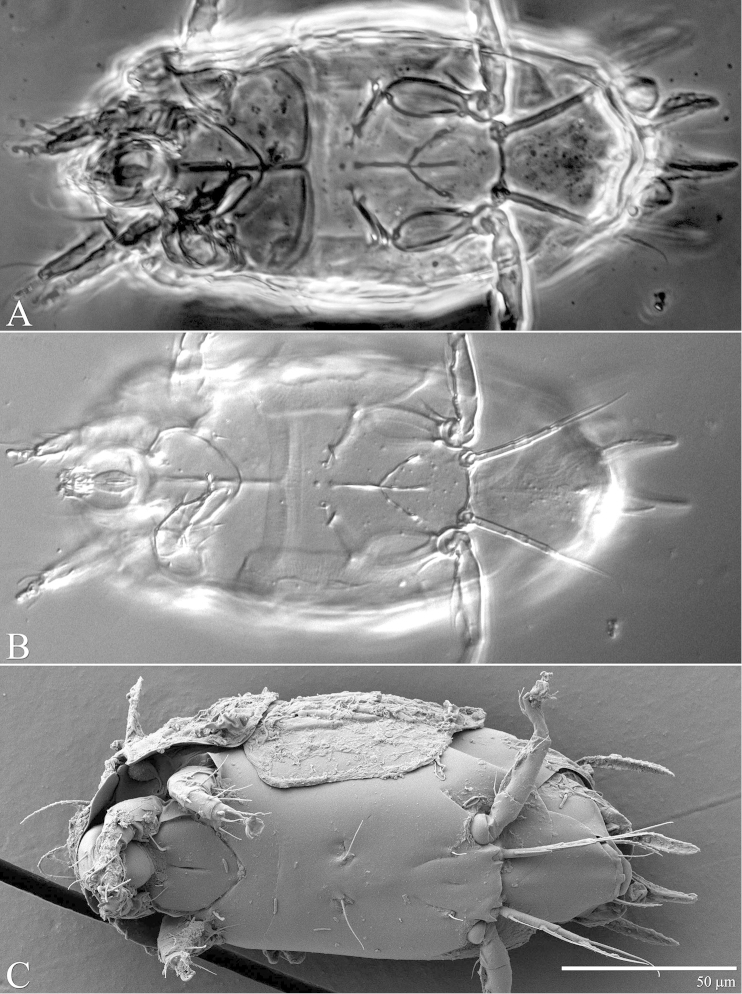
*Daidalotarsonemus oliveirai* sp. n. (female). Ventral micrographs. **A** phase contrast **B** differential interference contrast **C** low temperature scanning electron microscopy.

Idiosoma – venter (Figs 3–4): setae *1a* 6 (6–7), posteriad of apodemes 1; *2a* 9 (9), posterolaterad of apodemes 2; *3a* 14 (13–15) near anteriomedial margins of apodemes 3; *3b* 11 (11–12) on posterior margins of apodemes 4. Apodeme 1 conspicuous, fused to anterior end of prosternal apodeme. Apodeme 2 long and fused to the prosternal apodeme. Prosternal apodeme conspicuous from junction with apodeme 1 to the middle portion of sejugal apodeme. Sejugal apodeme uninterrupted, with a single median indentation. Apodeme 3 with a constriction near the anterior end, extending diagonally from proximity of base of seta *3a* to anterior margin of trochanter III; apodeme 4 extending diagonally from the middle of the poststernal apodeme to base of seta *3b*. Poststernal apodeme bifurcated anteriorly. Tegula wide 12 (11–13) and very short, 4 (4–5) (Fig. 7F); posterior margin slightly arched. Seta *ps* 12 (11–13) serrate. Ventral surface covered with tiny dimples (Fig. 7F).

**Figure 5.**
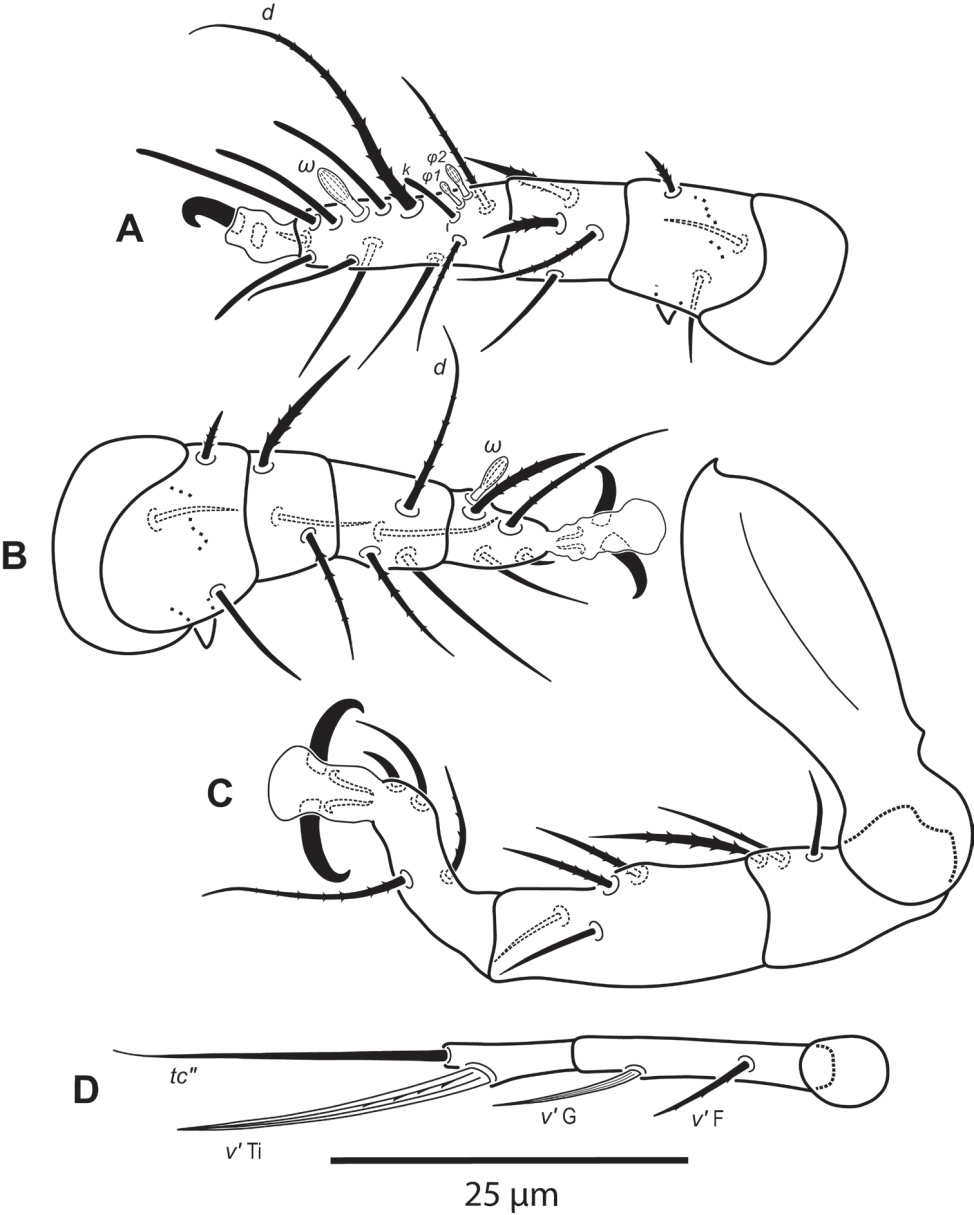
*Daidalotarsonemus oliveirai* sp. n. (female). **A** leg I **B** leg II **C** leg III **D** leg IV.

Legs (Fig. 5): lengths (measured from femur to tarsus): leg I 40 (39–42), leg II 37 (35–40), leg III 79 (78–80). Number of setae (solenidia in parentheses) on femur, genu, tibia and tarsus, respectively: leg I: 3-4-5(2)-7(1), leg II: 3-3-4-4(1), leg III: 1+2-4-4. Claws medium-sized (not reduced) and hooked. Empodia of the legs I, II and III about the same size or slightly smaller compared to the respective basal stalks. Tarsal solenidion *ω* of tibiotarsus I 6 (5–7), stout, wider medially. Sensory cluster of tibia I complete (Fig. 7A), solenidion *φ1* 3 (3–4), slender, capitate; solenidion *φ2* 4, robust, slightly capitate; famulus *k* 6; all inserted at approximately the same level. Seta *d* of tibia I 18 (18–19), serrate. Solenidion *ω* of tarsus II proximally inserted, 4 long, stout, wider medially (Fig. 7B). Seta *d* of tibia II 13 (13–14), serrate. Femorogenu IV 11 (14–15); tibiotarsus IV 8. Length of leg IV setae: *v*’ F 9, *v*’ G 11, *v*´ Ti 19 and *tc*” 24 (23–27); setae *v*’ Ti and *tc*” serrate; *v*’ Ti falcate.

#### Adult male

(3 specimens measured). Gnathosoma (Figs 10, 13 and 14A): subtriangular in ventral view, length 22 (21–23), maximum width 20 (19–20); dorsal apodeme distinct. Setae *dgs* 11 (10–12) and *vgs* 7 (7) smooth; Palps moderately long 9 (8–10), with 2 small subterminal setae and terminal projections. Pharynx fusiform, 15 (14–17) long and 7 (6–8) wide at widest region. Gnathosoma, idiosoma and legs covered with tiny dimples, each 0.3 (0.2–0.5) in diameter.

Idiosoma – dorsum (Figs 8–9): length 174 (170–178), maximum width 82 (80–84). Prodorsal shield trapezoidal. Length of dorsal setae: *v1* 30 (29–31), *v2* 24 (22–25), *sc1* 38 (37–40), *sc2* 24 (22–25), *c1* 21 (20–22), *c2* 26 (24–29), *d* 32 (30–34), *f* 14 (13–16). All setae setiform and serrate. Distances between dorsal setae: *v1*–*v1* 13 (12–14), *sc1*–*sc1* 34 (32–35), *sc2*–*sc2* 44 (43–46), *v1*–*sc2* 26 (25–27), *c1*–*c1* 75 (74–77), *c2*–*c2* 78 (76–80), *c1*–*c2* 44 (43–47), *d*–*d* 45 (44–47), *f*–*f* 22 (20–23). Seta *sc2* laterad and slightly posterior to *sc1*; seta *c1* closer to *d* than to *c2*, anterolateral to the latter.

Idiosoma – venter (Figs 10–11): setae *1a* 6 (5–6) posteriad of apodemes 1; setae *2a* 7 (7–8) located in the center of coxisternal plate 2; seta *3a* 12 (11–13) located near anterior end of apodeme 3; and seta *3b* 12 (10–14) located near middle of apodeme 4. Apodeme 1 fused to anterior end of prosternal apodeme; apodeme 2 not fused to prosternal apodeme. Prosternal apodeme conspicuous between coxisternal plates I but thin between coxisternal plates II, extending close to sejugal apodeme. Sejugal apodeme conspicuous. Lines of fusion between coxae III and IV with venter of idiosoma mostly conspicuous (apodemes 3 and 4, poststernal apodeme and connecting apodeme between apodemes 3 and 4); connecting apodemes between apodemes 4 and poststernal diffuse.

Legs (Fig. 12): lengths (measured from femur to tarsus): leg I 63 (62–65), leg II 59 (57–61), leg III 81 (79–83), leg IV 83 (81–84). Number of the setae (solenidia in parentheses) on femur, genu, tibia and tarsus, respectively: leg I: 4-4-6(2)-9(1), leg II: 3-3-4-4(1), leg III: 1-3-4-3. Claws medium-sized (not reduced) and hooked. Empodia of the legs I, II and III about the same size or slightly smaller compared to the respective basal stalks. Solenidion *ω* of tarsus I 4 (3–5), stout, wider medially. Sensory cluster of tibia I composed of *φ1* 3 (3), *φ2* 4 (4–5) and famulus *k* 4 (4), all inserted at approximately same level (Fig. 14B). Seta *d* of tibia I 27 (26–30), serrate. Solenidion *ω* of tarsus II proximally inserted 4 (4–5) long, stout, wider medially (Fig. 14C). Seta *d* of tibia II 23 (21–24), serrate. Trochanter IV slightly wider than long, seta *v*’ 13 (12–14), smooth. Femorogenu IV 41 (40–43) long and 17 (16–19) wide at *v*’ F level; anterior margin convex, posterior margin slightly convex at proximal third, with a serrate-like projection between these margins. Seta *v*’ F 9 (8–10), serrate. Setae *v*’ G 17 (16–18) and *l*” G 12 (11–13), smooth. Tibia IV 24 (22–26) long; solenidion *φ* 7 (6–8); seta *v*’ Ti 28 (27–31), serrate. Tarsus IV short, bearing 3 smooth setae of the following length: *tc*” 4 (4–5), *pv*” 6 (5–7) and *u*’ 5 (4–6). Claw well developed (Fig. 14D).

#### Type material.

Holotype female, allotype male, 6 paratype females and 2 paratype males from *Theobroma cacao* L., 1 paratype female from *Annona muricata* L. and 2 paratype females from *Spondias purpurea* L., 14°47'45"S; 39°10'18"W, Ilhéus, State of Bahia, Brazil, 10/IX/2012, A.C. Lofego and J.M. Rezende. Holotype, allotype, 7 paratype females and 2 paratype males are deposited at DZSJRP and 2 paratype females are deposited at USNM.

#### Etymology.

The species name *oliveirai* is in honor of Dr. Anibal Ramadan Oliveira (UESC - Universidade Estadual Santa Cruz from Ilhéus-BA) for his contribution to study of mites and for all his support during the samplings in the region.

**Figure 6.**
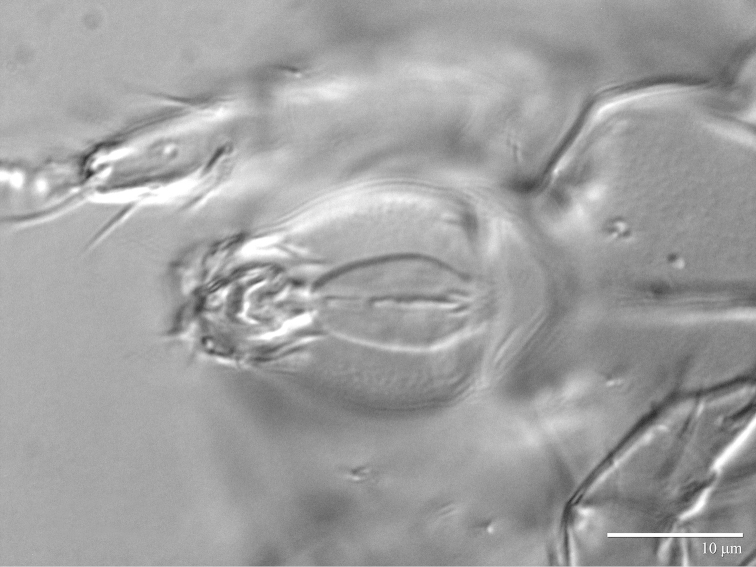
*Daidalotarsonemus oliveirai* sp. n. (female). Detail of the gnathosoma.

**Figure 7.**
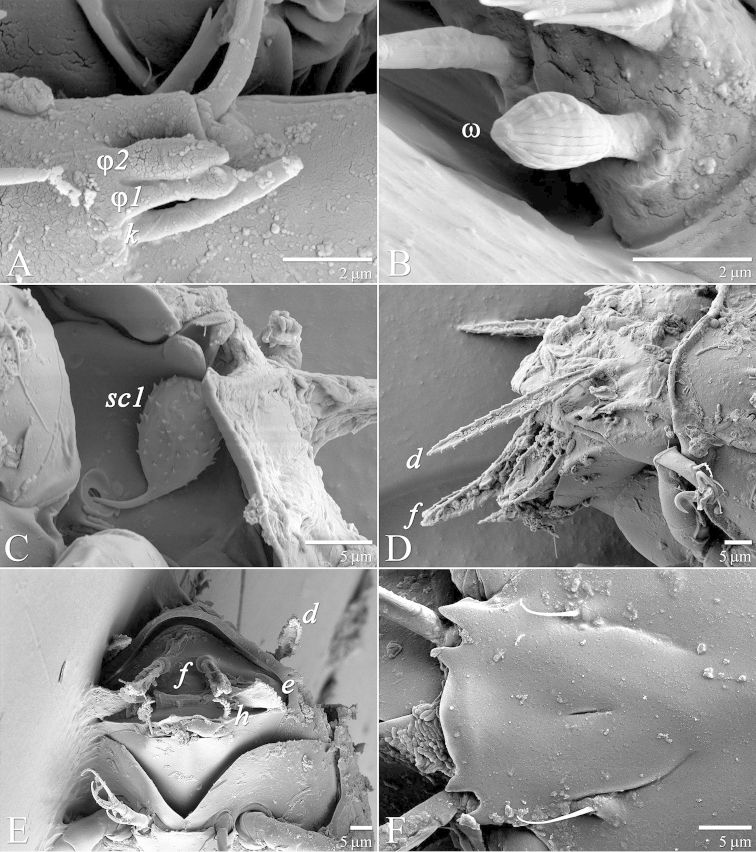
*Daidalotarsonemus oliveirai* sp. n. (female). **A** sensorial cluster of tibia I **B** Solenidion *ω* of tarsus II **C** Bothridial seta *sc1* **D** lateral view of the setae *d*, *e* and *f* **E** posterior view of the setae *d*, *e* and *f* **F** detail of the tegula.

**Figure 8.**
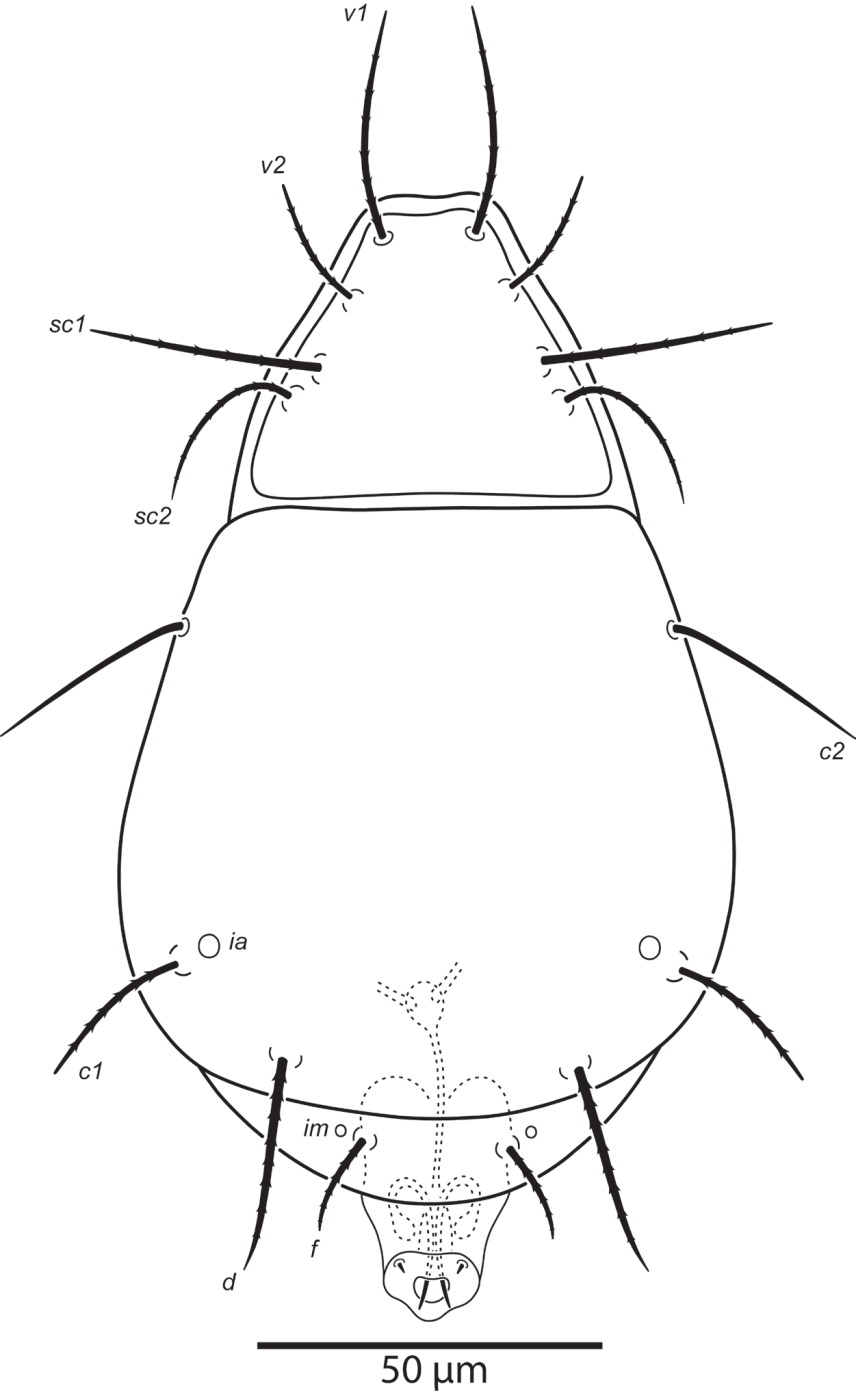
*Daidalotarsonemus oliveirai* sp. n. (male). Dorsal surface.

**Figure 9.**
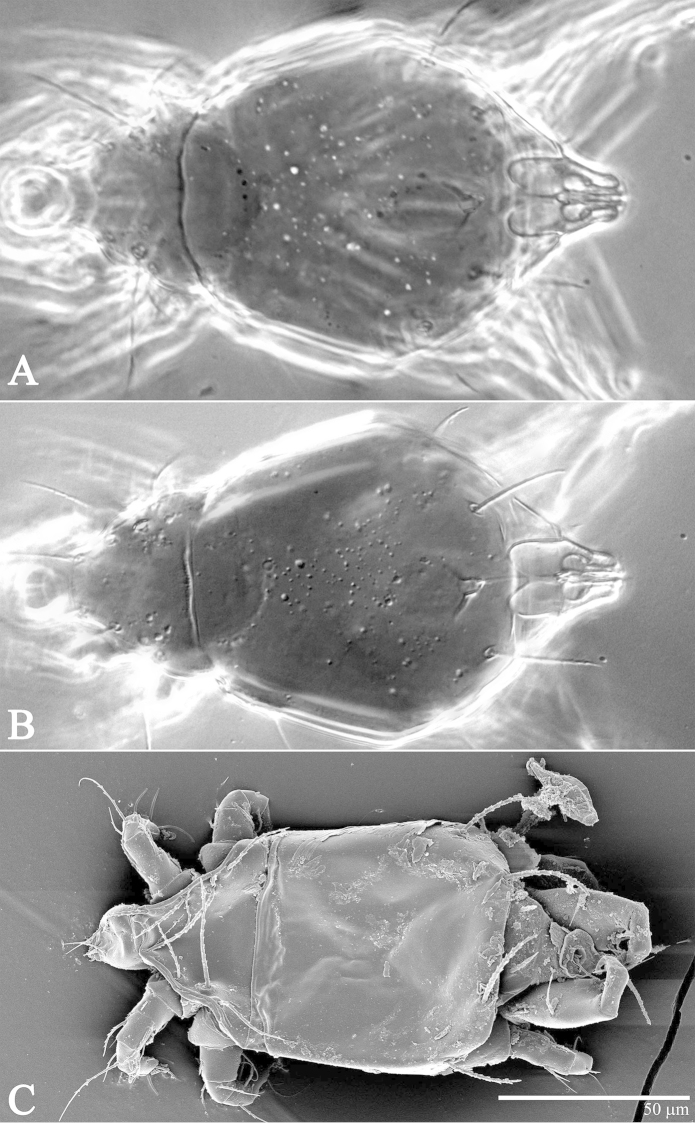
*Daidalotarsonemus oliveirai* sp. n. (male). Dorsal micrographs. **A** phase contrast **B** differential interference contrast **C** low temperature scanning electron microscopy.

**Figure 10.**
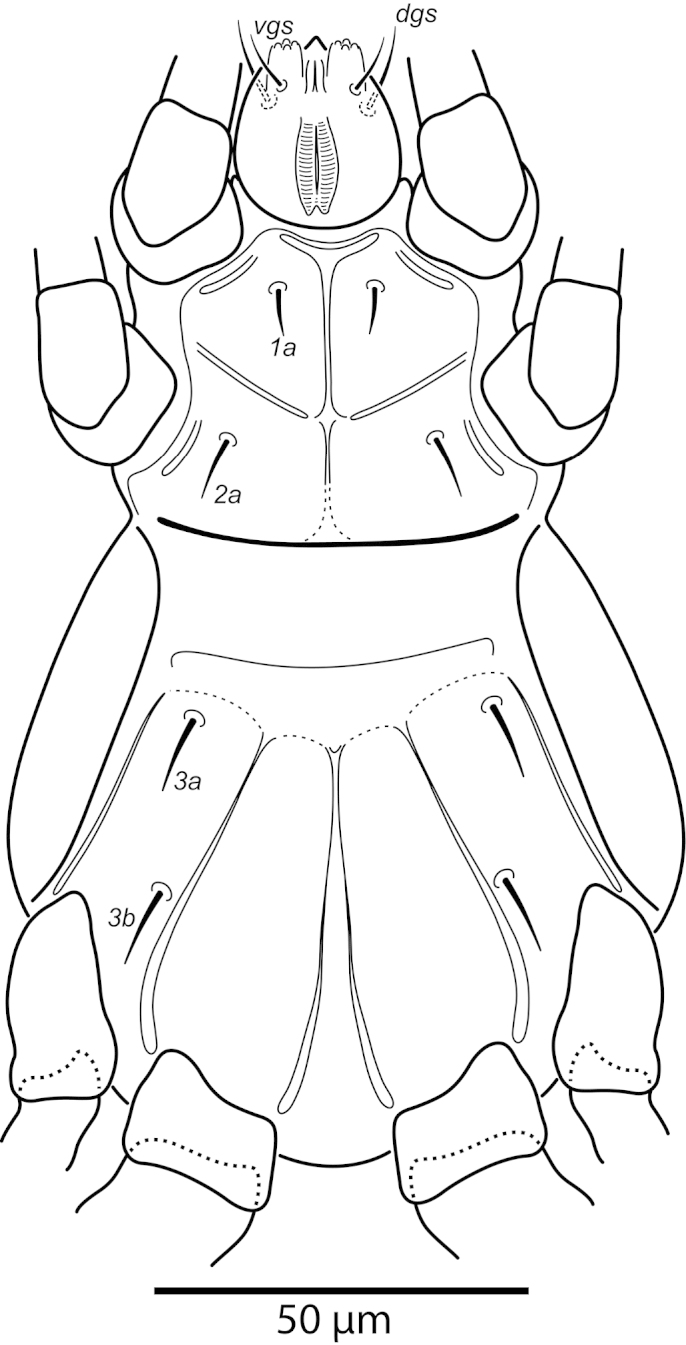
*Daidalotarsonemus oliveirai* sp. n. (male). Ventral surface.

**Figure 11.**
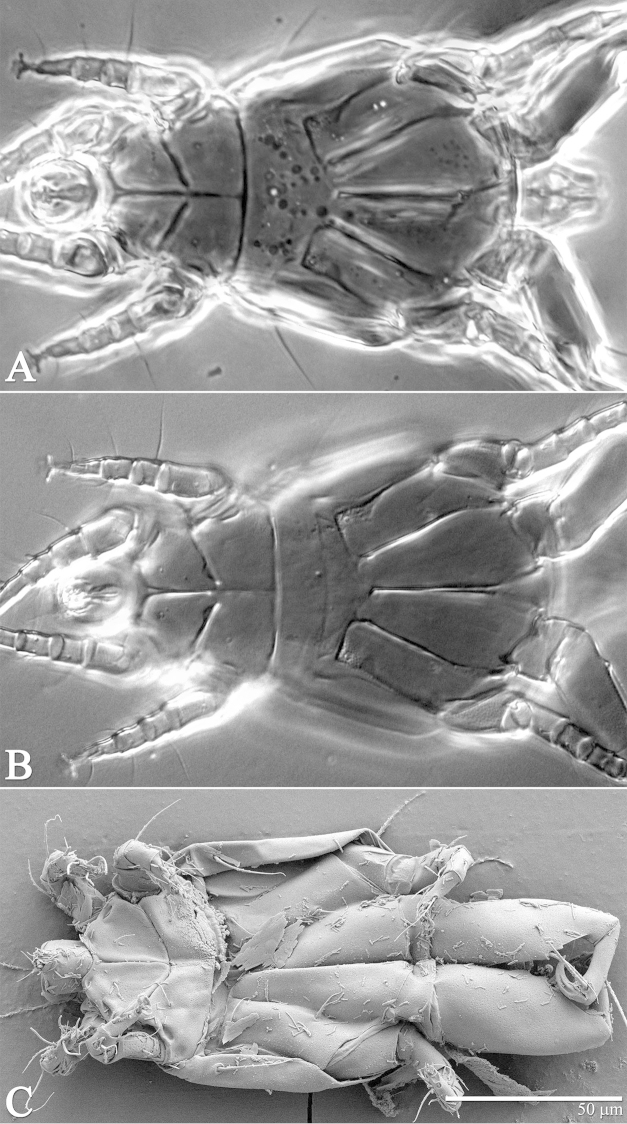
*Daidalotarsonemus oliveirai* sp. n. (female). Ventral micrographs. **A** phase contrast **B** differential interference contrast **C** low temperature scanning electron microscopy.

**Figure 12.**
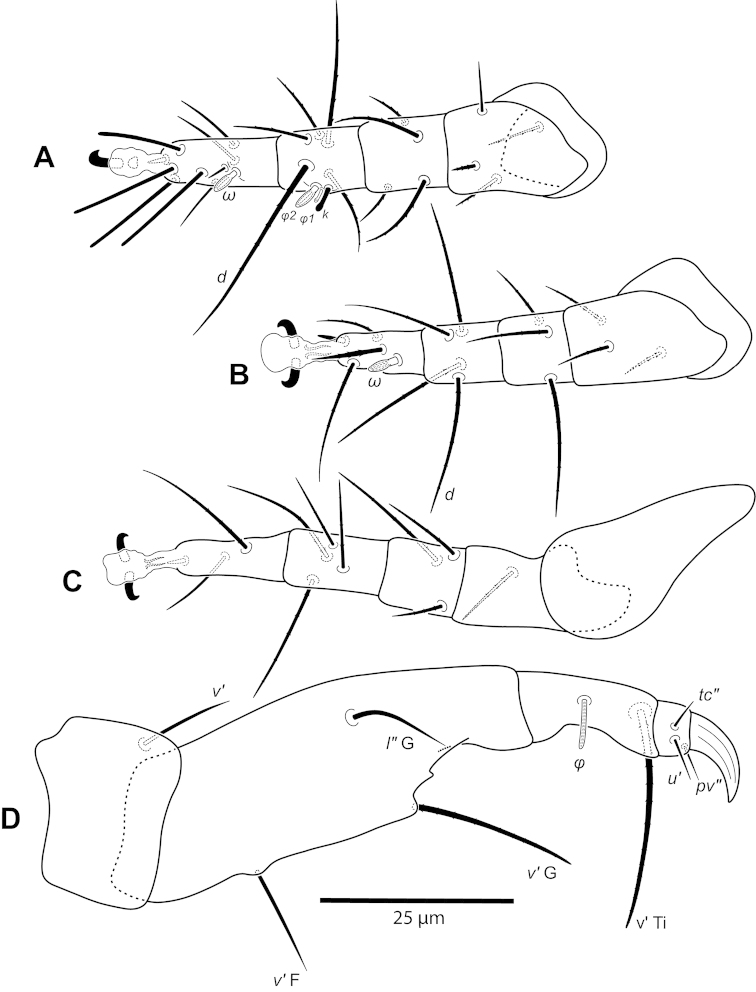
*Daidalotarsonemus oliveirai* sp. n. (male). **A** leg I **B** leg II **C** leg III **D** leg IV.

**Figure 13.**
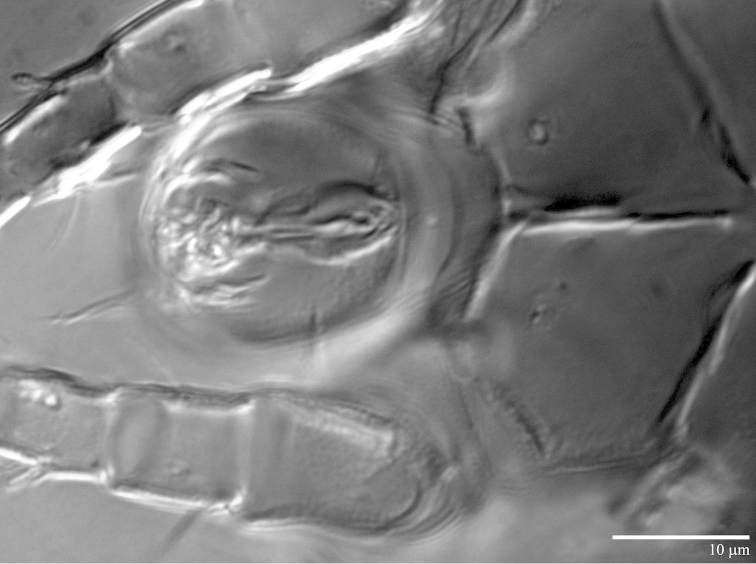
*Daidalotarsonemus oliveirai* sp. n. (male). Detail of the gnathosoma.

**Figure 14.**
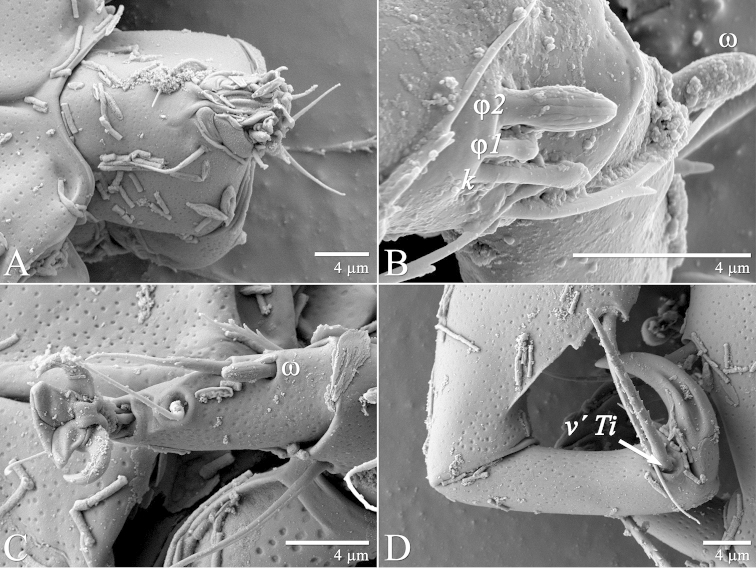
*Daidalotarsonemus oliveirai* sp. n. (male). **A** gnatosoma **B** sensorial cluster of tibia I **C** tarsus II **D** part of tibia and tarsus IV.

### Excelsotarsonemus caravelis

Taxon classificationAnimaliaTrombidiformesTarsonemidae

Rezende, Lofego & Ochoa sp. n.

http://zoobank.org/101417BE-223C-4746-9107-05528AD1A7F8

**Figure 15.**
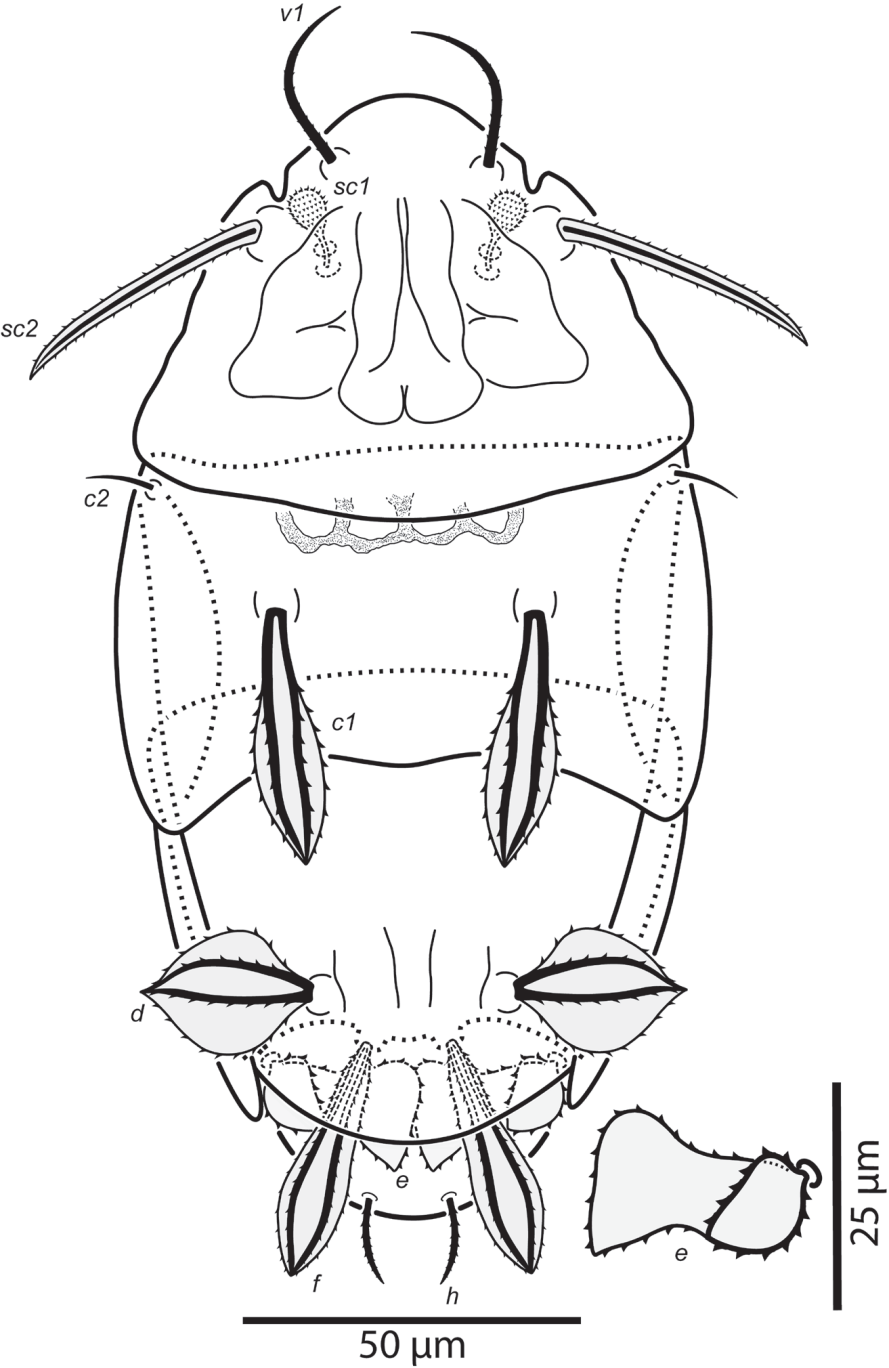
*Excelsotarsonemus caravelis* sp. n. (female). Dorsal surface.

**Figure 16.**
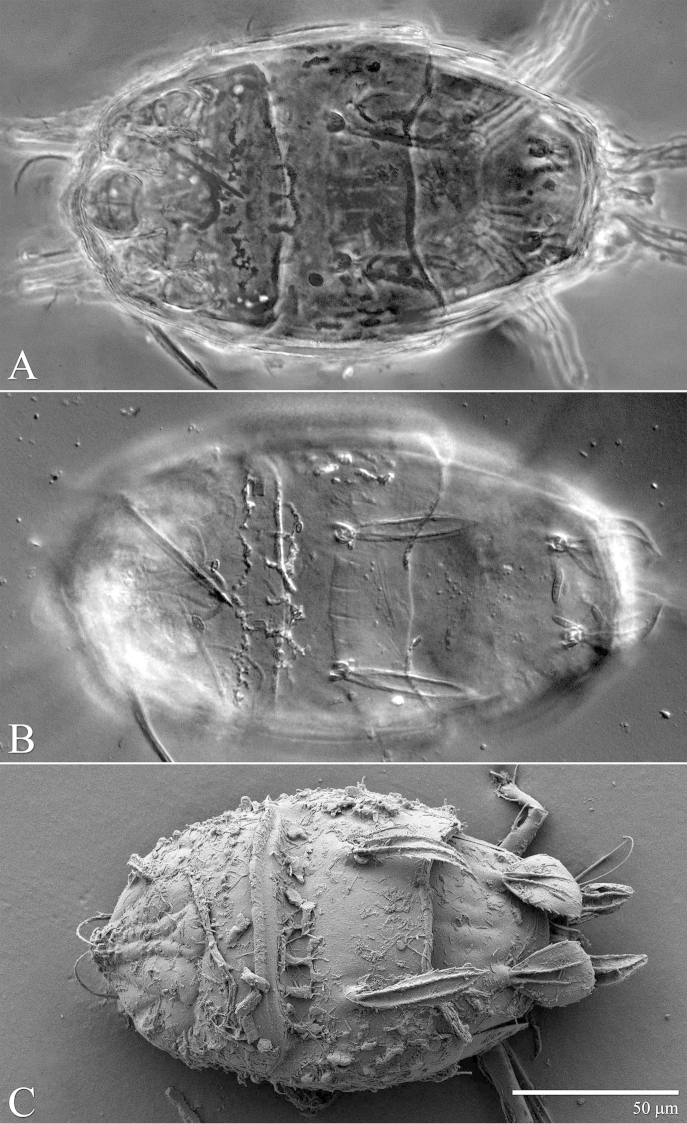
*Excelsotarsonemus caravelis* sp. n. (female). Dorsal micrographs. **A** phase contrast **B** differential interference contrast **C** low temperature scanning electron microscopy.

**Figure 17.**
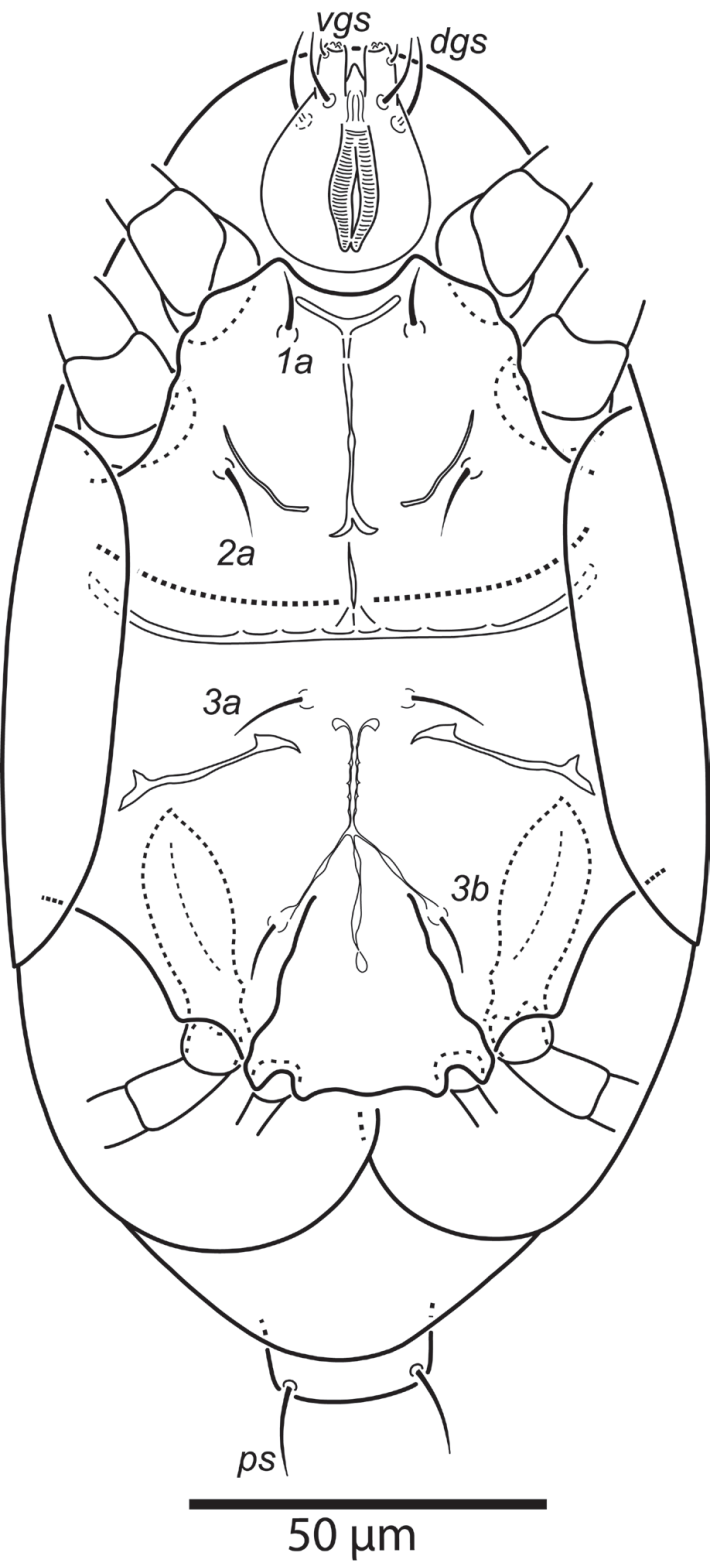
*Excelsotarsonemus caravelis* sp. n. (female). Ventral surface.

**Figure 18.**
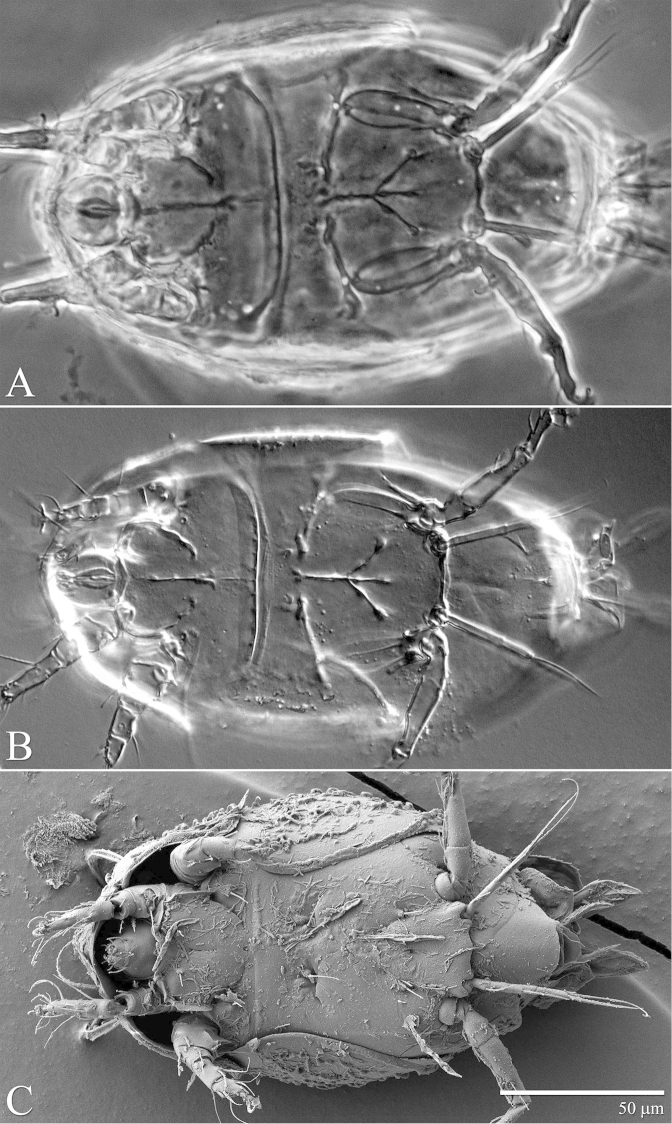
*Excelsotarsonemus caravelis* sp. n. (female). Ventral micrographs. **A** phase contrast **B** differential interference contrast **C** low temperature scanning electron microscopy.

**Figure 19.**
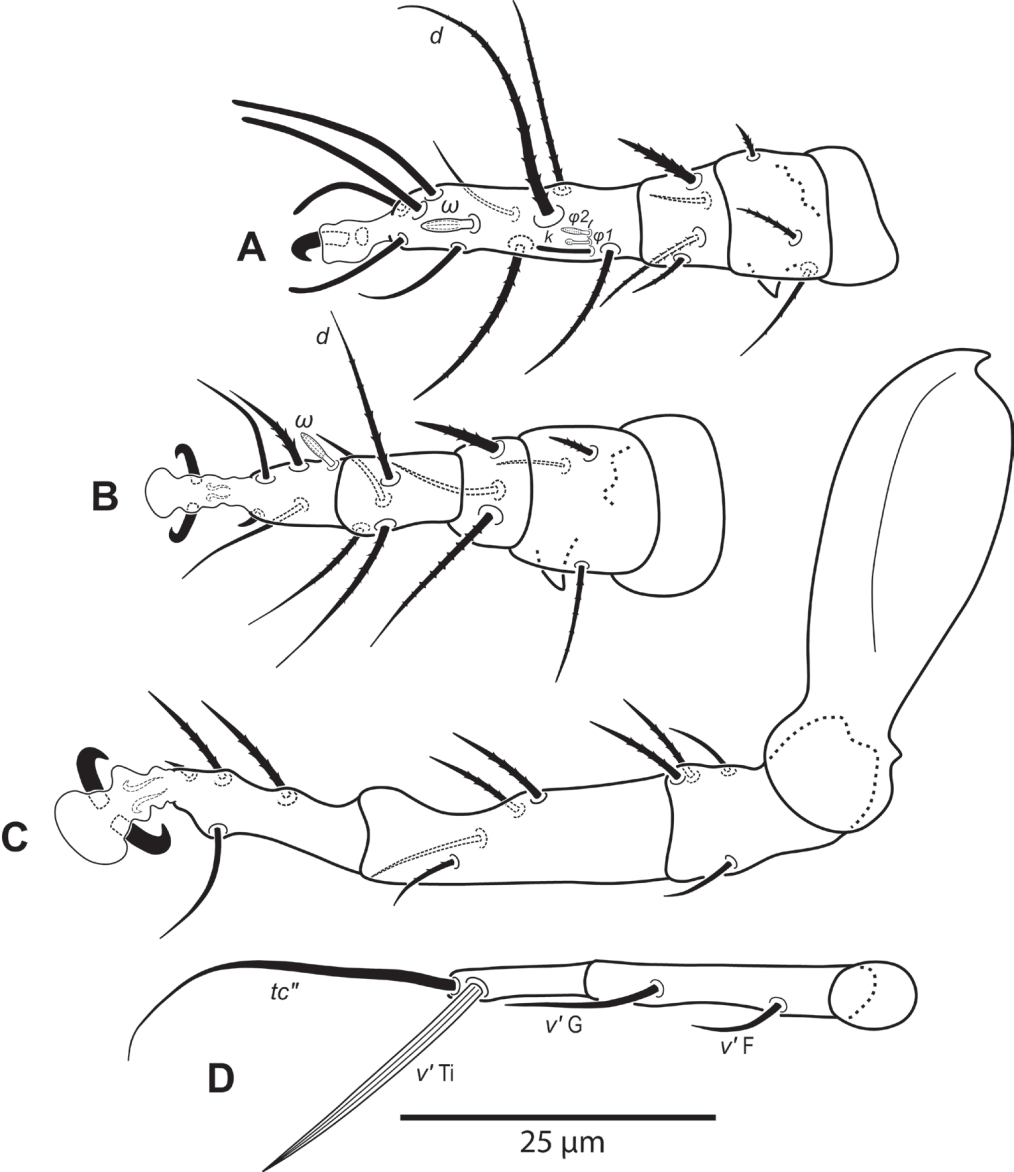
*Excelsotarsonemus caravelis* sp. n. (female). **A** leg I **B** leg II **C** leg III **D** leg IV.

**Figure 20.**
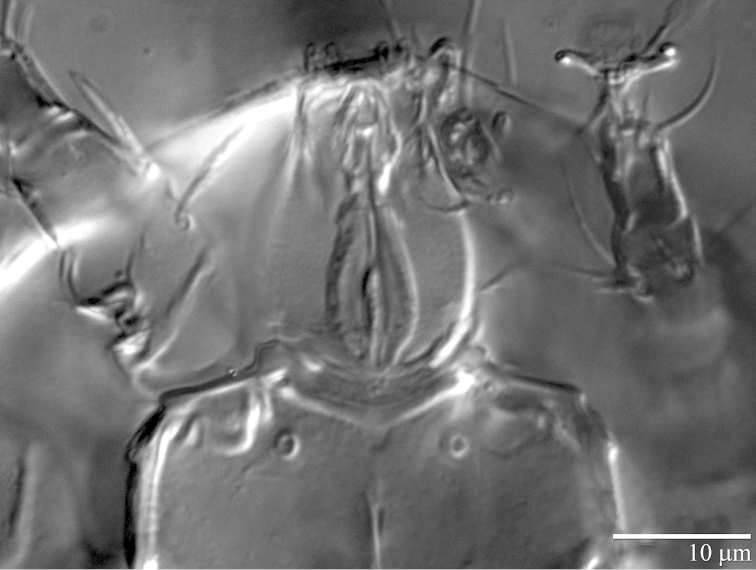
*Excelsotarsonemus caravelis* sp. n. (female). Detail of the gnathosoma.

**Figure 21.**
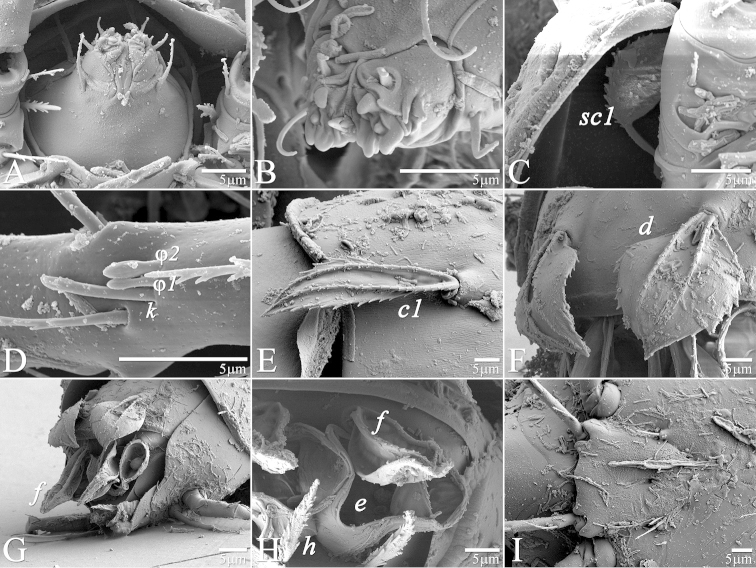
*Excelsotarsonemus caravelis* sp. n. (female). **A** gnatosoma **B** detail of the palps **C** Bothridial seta *sc1* **D** sensorial cluster of tibia I **E** seta *c1* **F** seta *d* **G** tergites **D, E, F, H** and posterior setae **H** setae *e*, *f* and *h* **I** tegula.

Figs 15 , 16 , 17 , 18 , 19 , 20 , 21

#### Diagnosis.

Females of this species resemble those of *Excelsotarsonemus kimhansenae* Ochoa & OConnor because of the shape of dorsal setae *v1*, *sc2*, *c1* and *c2*, and the ornamentation pattern on the prodorsum; but they are distinguished by the asymmetric shape of setae *e* and the U-shaped cerotegument accumulation between prodorsum and tergite C in *Excelsotarsonemus caravelis* sp. n., whereas setae *e* are orbicular and smooth and tergite C surface is smoother in *Excelsotarsonemus kimhansenae*. The accumulation of the cerotegument between the tergites was easily noticed in all microscopy techniques used (Fig. 16), and it is being considered a taxonomic feature, useful for distinguishing these species.

#### Adult female

(5 specimens measured). Gnathosoma (Figs 17, 20, 21A-B): completely covered by prodorsum. Subtriangular in ventral view, length 22 (21–24), maximum width 17 (16–19); dorsal apodeme distinct. Setae *dgs* 7 (7–8) and *vgs* 5 (5–6) smooth; palps moderately short 6–8 (7), with 2 small subterminal setae and terminal projections. Pharynx fusiform, 15 (15–16) long and 6 (6) wide at maximum width. Gnathosoma, idiosoma and legs covered with tiny dimples, each 0.3 (0.2–0.5) in diameter.

Idiosoma – dorsum (Figs 15–16): length 167 (166–168), width at level of *c1* 86 (84–90); prodorsal shield normally covering entire gnathosoma, with three external humps, broader proximally, central area with an inverted Y-shaped pattern. Stigma near lateral notch of the prodorsal shield, equidistant to the *v1* and *sc2* setal bases. Entire dorsum covered with cerotegument with a U-shaped cerotegument accumulating between the prodorsum and tergite C (Fig. 16). Tergite D with irregular bumps near setae *d.* Lengths of the setae: *v1* 29 (27–31), *sc1* 16 (14–18) (Fig. 21C), *sc2* 47 (45–49), *c1* 40 (40–41) (Fig. 21E), *c2* 9 (8–10), *d* 30 (28–32) (Fig. 21F), *e* 16 (16–17), *f* 36 (35–38) and *h* 13 (11–16). Maximum width of expanded setae: *sc2* 3 (3–4), *c1* 11 (11–12), *d* 22 (21–23), *e* 32 (31–33) and *f* 12 (11–13). All setae serrate, except for *c2* which is smooth. Bothridial setae *sc1* capitate with tiny spines; *sc2* linear with a strong central furrow; setae *c1* lanceolate, *d* ovate and *f* oblanceolate with serrate central veins; *e* each totally asymmetric (Figs 21G-H). Distances between dorsal setae: *v1*–*v1* 26 (24–29), *sc2*–*sc2* 46 (45–48), *v1*–*sc2* 15 (14–16), *c1*–*c1* 43 (41–45), *c2*–*c2* 89 (85–96), *c1*–*c2* 36 (34–38), *d*–*d* 27 (27–28), *f*–*f* 11 (9–13), *e*–*f* 12 and *h*–*h* 5 (4–7). Seta *sc2* located anteriorly to *sc1*. Dorsal cupules not easily seen.

Idiosoma – venter (Figs 17–18): seta *1a* 6 (6–7), posteriad of apodemes 1; *2a* 9 (9–10), posterolaterad of apodemes 2; *3a* 11 near anteriomedial margins of apodemes 3; *3b* 8 (8–9) on posterior margins of apodemes 4. Apodeme 1 conspicuous and fused to anterior end of prosternal apodeme. Apodeme 2 short and not fused to prosternal apodeme. Prosternal apodeme conspicuous from junction with apodeme 1 near middle of sejugal apodeme portion. Sejugal apodeme uninterrupted with several small indentations. Apodeme 3 with a constriction near anterior end, extending diagonally from proximity of base of seta *3a* to anterior margin of trochanter III; apodeme 4 extending diagonally from the middle of the poststernal apodeme to base of seta *3b*. Poststernal apodeme bifurcated anteriorly. Tegula wide 16 (15–17) and very short, 4 (4–5) (Fig. 21I), posterior margin slightly arched. Setae *ps* 17 (16–19) smooth.

Legs (Fig. 19): lengths (measured from femur to tarsus): leg I 42 (42–43), leg II 40 (39–41), leg III 92 (89–95), leg IV 32 (31–35). Number of setae (solenidia in parentheses) on femur, genu, tibia and tarsus, respectively: leg I: leg I: 3-4-5(2)-7(1), leg II: 3-3-4-4(1), leg III: 2+2-4-4. Claws medium-sized (not reduced) and hooked. Empodia of the legs I, II and III about the same size or slightly smaller compared to the respective basal stalks. Tarsal solenidion *ω* of tibiotarsus I 4 (4–5), stout, wider medially. Sensory cluster of tibia I complete (Fig. 21D), solenidion *φ1* 4 (4–5), slender, capitate; solenidion *φ2* 3, robust, slightly capitate; famulus *k* 6; all those inserted at approximately in the same level. Seta *d* of tibia I 23 (22–24), serrate. Solenidion *ω* of tarsus II proximally inserted, 4 long, stout, wider medially. Seta *d* of tibia II 17 (17–18), serrate. Femorogenu IV 18 (16–20); tibiotarsus IV 9 (9–10). Length of leg IV setae: *v*’ F 8 (8–9), *v*’ G 10 (10–11), *v*’ Ti 23 (22–24) and *tc*” 31 (29–33); setae *v*’ Ti and *tc*” smooth; *v*’ Ti falcate.

#### Adult male.

Unknown.

#### Type material.

Holotype female and 4 paratype females on *Theobroma cacao* L., 14°47'45"S; 39°10'18"W, Ilhéus, State of Bahia, Brazil, 10/IX/2012, A.C. Lofego and J.M. Rezende. Holotype and 3 paratypes are deposited in the DZSJRP and 1 paratype is deposited in the USNM.

#### Etymology.

The region where this mite was found is the same place as the first Portuguese explorers arrived in Brazil, at the end of 15^th^ century. On their trip, they used caravels, which had big sails. The name *caravelis* is used because several dorsal setae of this mite species are held in the upright position resembling those sails.

#### Note.

Setae *f* has a unique modification as it is oblanceolate dorsal view, with four faces attached by the main vein, giving a deep concavity at either site, with a central furrow dorsally shoe-like; all margins serrate (Fig. 21H). Similar setal complex modification has been observed in *Excelsotarsonemus mariposa* (setae *d*, *f* and *e*) and other *Excelsotarsonemus* and *Daidalotarsonemus* species under DIC. However, it is under the LT-SEM that we can understand their complexity.

**Figure 22.**
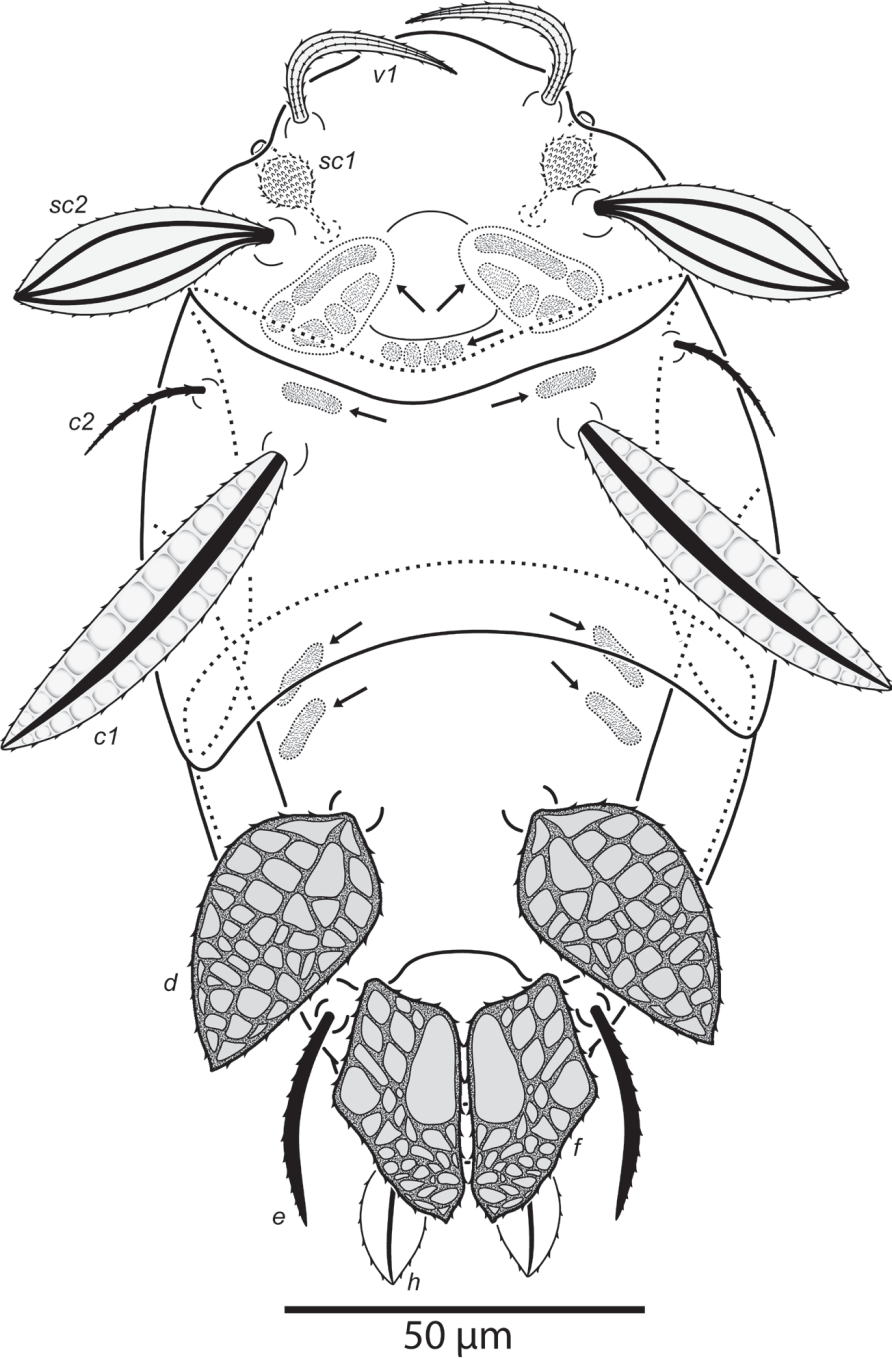
*Excelsotarsonemus tupi* sp. n. (female). Dorsal surface (arrows indicate muscle attachments present over the body).

### Excelsotarsonemus tupi

Taxon classificationAnimaliaTrombidiformesTarsonemidae

Rezende, Lofego & Ochoa sp. n.

http://zoobank.org/B37CCDE2-2748-47BD-B46B-801AD0F22D6F

**Figure 23.**
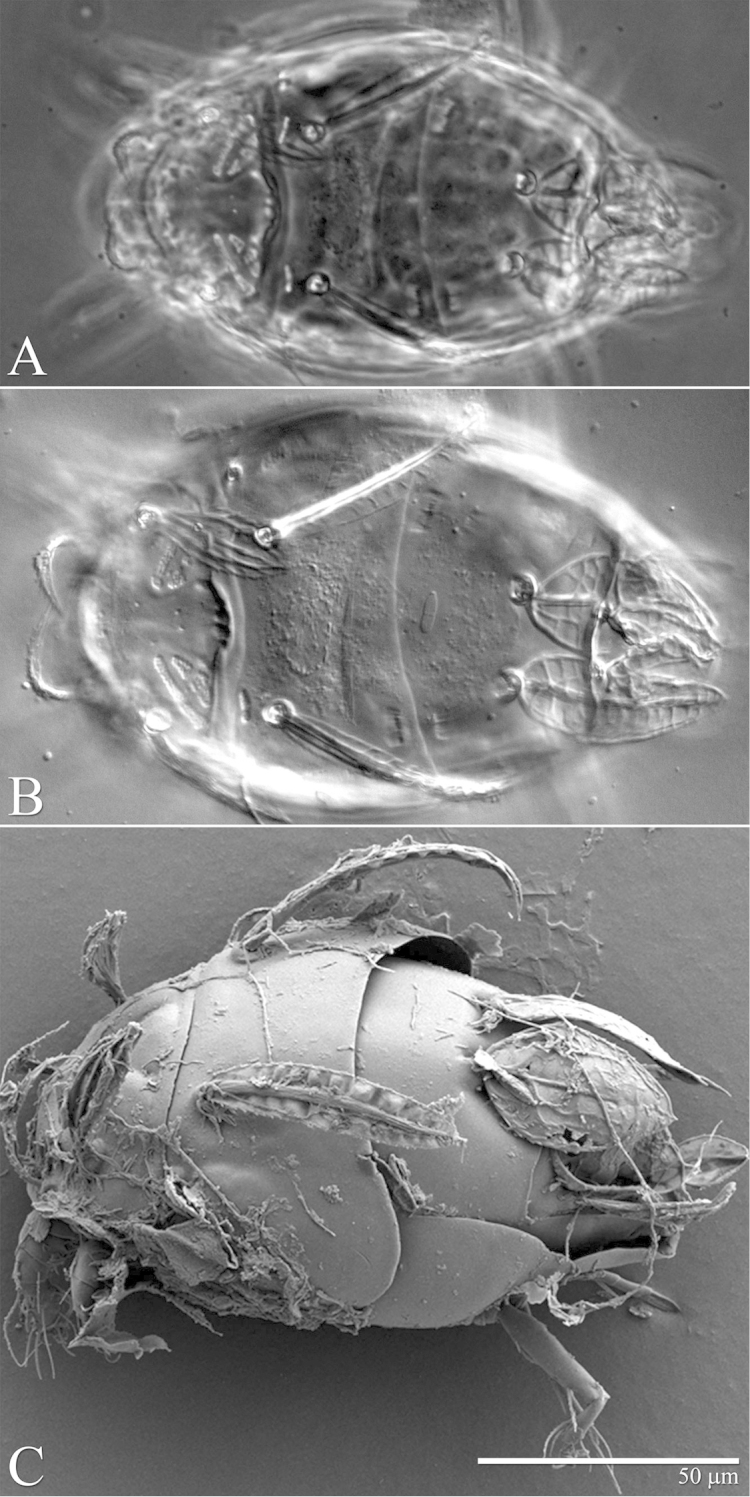
*Excelsotarsonemus tupi* sp. n. (female). Dorsal micrographs. **A** phase contrast **B** differential interference contrast **C** low temperature scanning electron microscopy.

**Figure 24.**
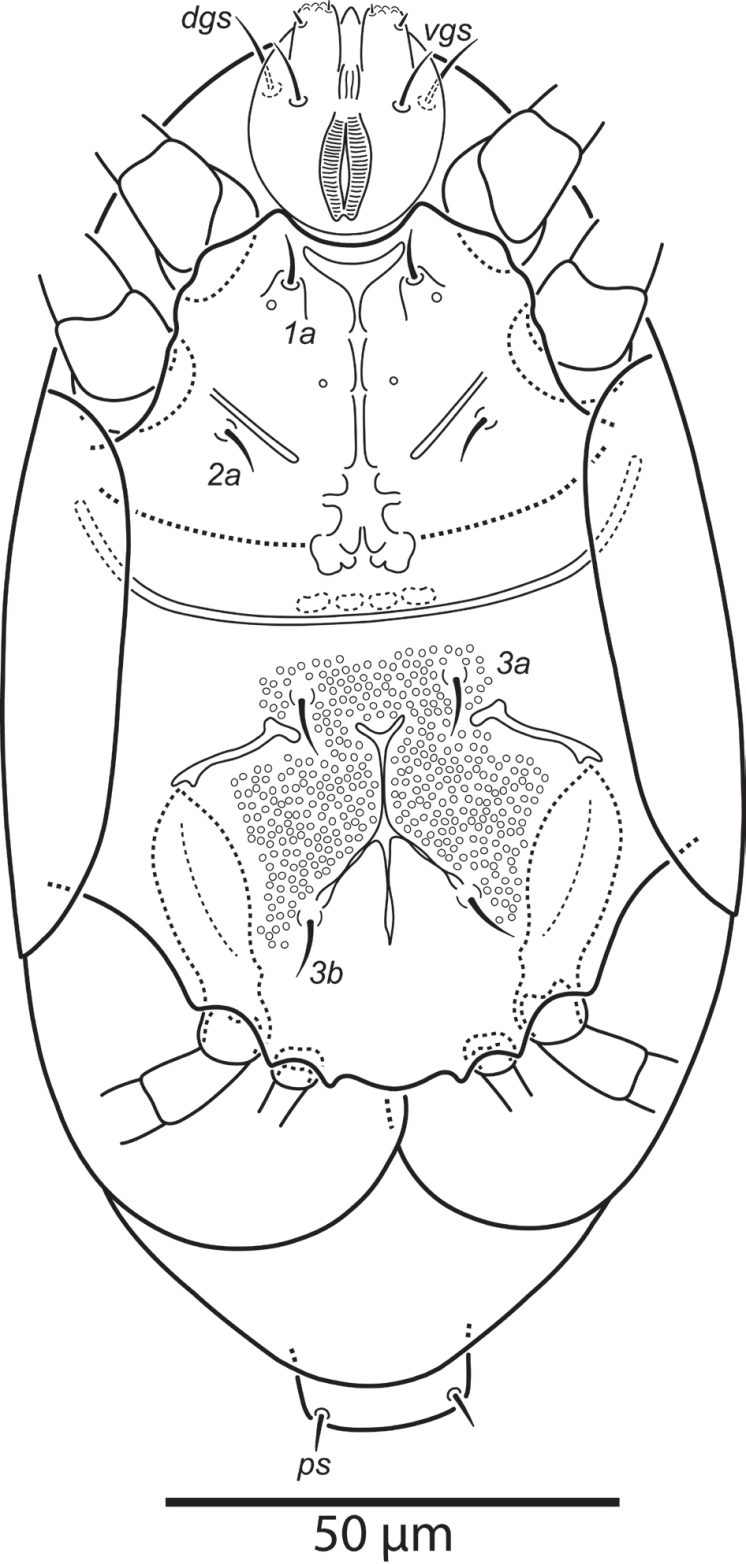
*Excelsotarsonemus tupi* sp. n. (female). Ventral surface.

**Figure 25.**
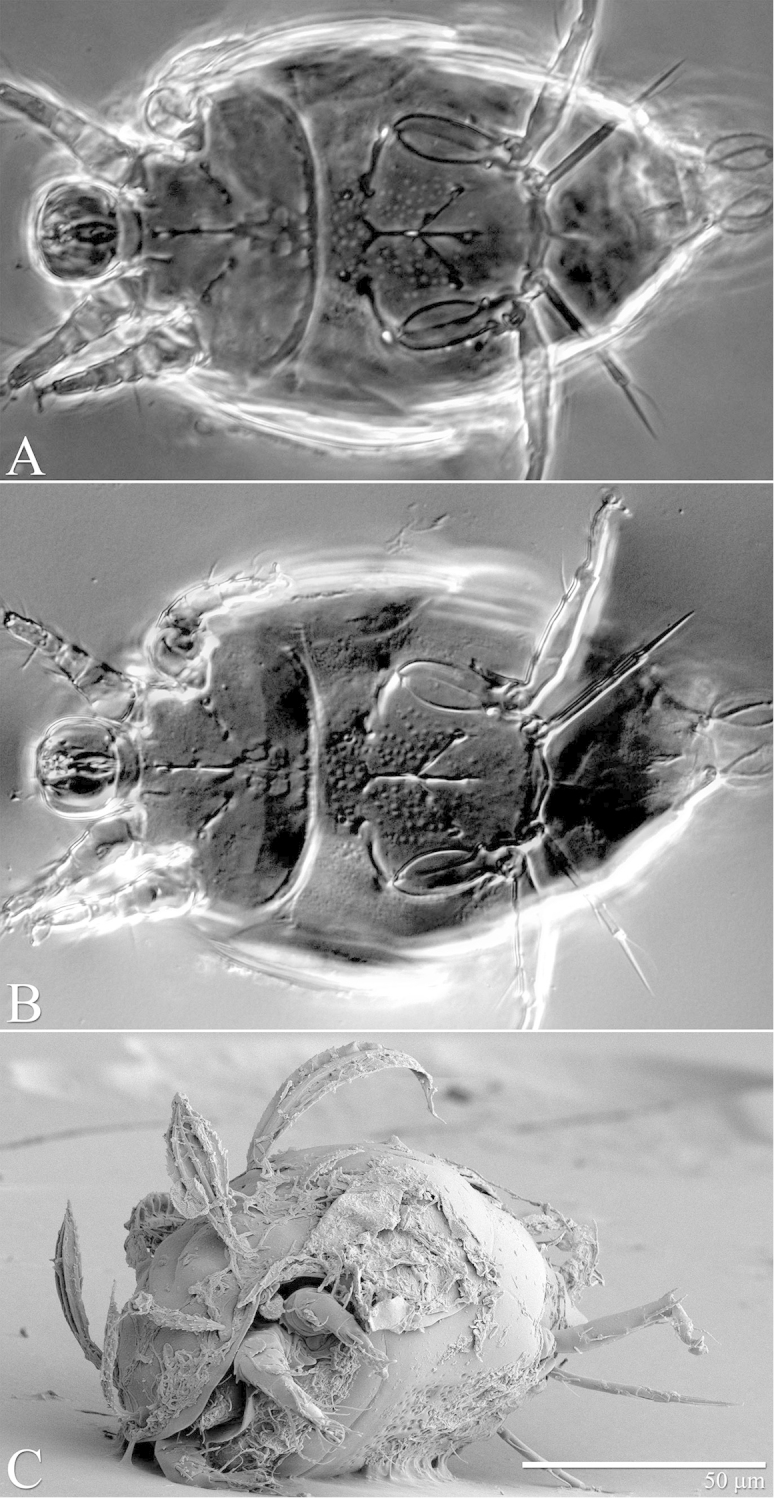
*Excelsotarsonemus tupi* sp. n. (female). Ventral micrographs. **A** phase contrast **B** differential interference contrast **C** low temperature scanning electron microscopy.

**Figure 26.**
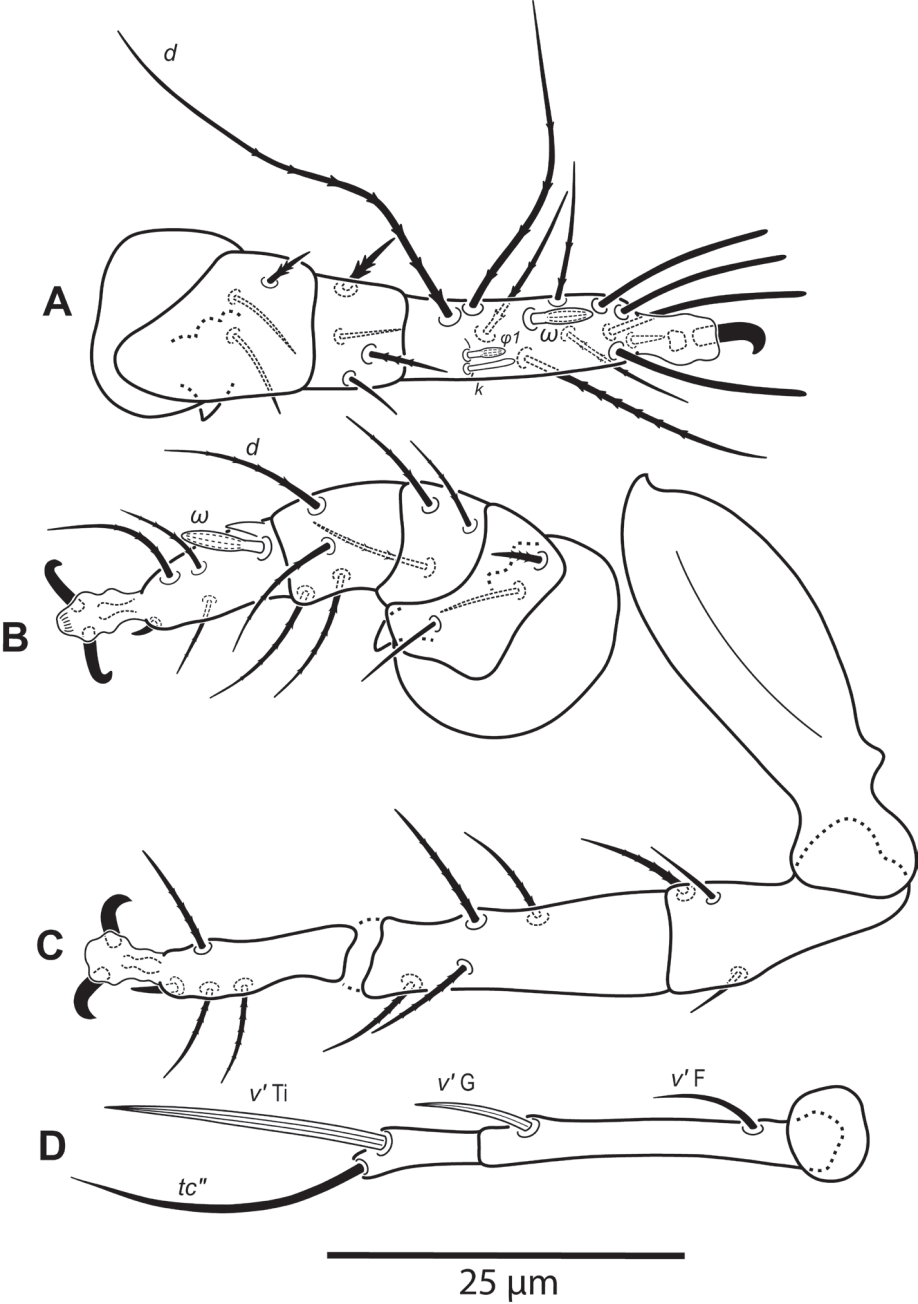
*Excelsotarsonemus tupi* sp. n. (female). **A** leg I **B** leg II **C** leg III **D** leg IV.

**Figure 27.**
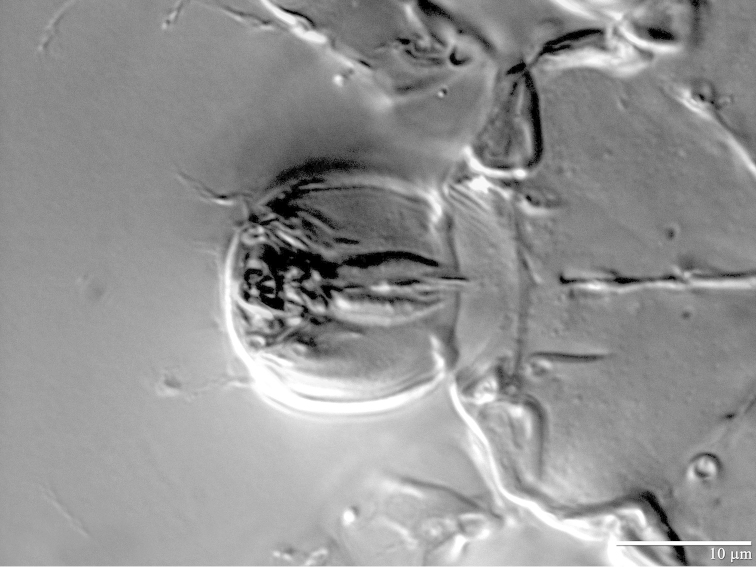
*Excelsotarsonemus tupi* sp. n. (female). Detail of the gnathosoma.

**Figure 28.**
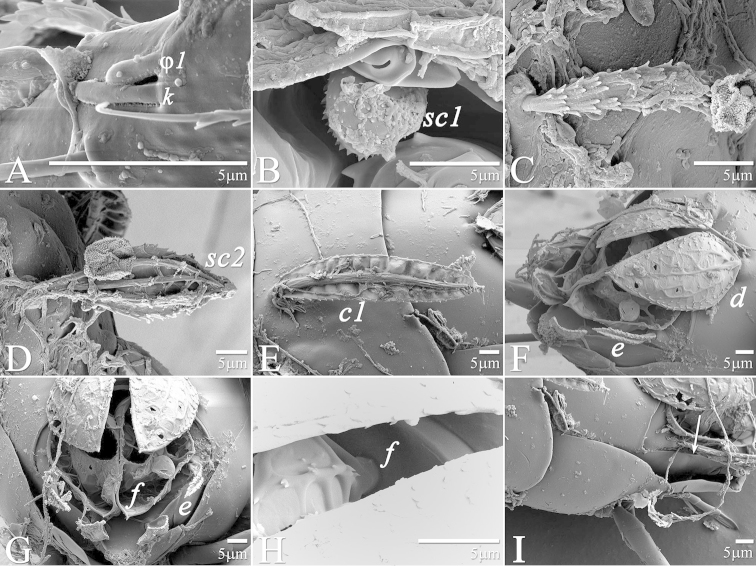
*Excelsotarsonemus tupi* sp. n. (female). **A** sensorial cluster of tibia I **B** Bothridial seta *sc1* and stigma opening **C** seta *v1* **D** seta *sc2* **E** seta *c1* **F** lateral view of the setae *d*, *e*, *f* and *h* **G** posterior view of the setae *d*, *e*, *f* and *h* **H** insertion of seta *f* **I** posterior view of tergites E, F and H (which is indicated by an arrow).

Figs 22 , 23 , 24 , 25 , 26 , 27 , 28

#### Diagnosis.

Females of this species resemble those of *Excelsotarsonemus kaliszewskii* Ochoa & Naskręcki (Ochoa et al. 1995) because of the similar shape of setae *sc2*, *c1* and *d.* However, setae *c2* and *e* of *Excelsotarsonemus tupi* sp. n. are setiform-like, while in *Excelsotarsonemus kaliszewskii* these setae are falcate and elongate. In addition, the humps on the prodorsum and the muscle attachments of tergite D are very different in shape between these two species, being more ornate and prominent in *Excelsotarsonemus kaliszewskii*.

#### Adult female

(3 specimens measured). Gnathosoma (Figs 24 and 27): completely covered by the prodorsum. Subtriangular in ventral view, length 21 (21–22), maximum width 17 (16–19); dorsal apodeme distinct. Setae *dgs* 8 (7–9) and *vgs* 4 (4–5) smooth; Palps moderately short 6–8 (7), with 2 small subterminal setae and terminal projections. Pharynx fusiform, 15 (14–16) long and 8 (7–9) wide at maximum width region. Gnathosoma, idiosoma and legs covered with tiny dimples, each around 0.3 (0.2–0.5) in diameter.

Idiosoma – dorsum (Figs 22–23): length 175 (171–179), width at level of *c1* 94 (93–95); prodorsal shield covering gnathosoma. Entire dorsum covered with cerotegument. Stigma inserted proximally at the lateral notch of the prodorsal shield, near the base of setae *v1*. Prodorsum, tergites C and D with distinct muscle attachments, visible with DIC and PC optic microscopes. Lengths of the setae: *v1* 23 (22–25) (Fig. 28C), *sc1* 15 (15–16) (Fig. 28B), *sc2* 44 (43–47) (Fig. 28D), *c1* 46 (44–49) (Fig. 28E), *c2* 14 (11–17), *d* 32 (31–34), *e* 26 (25–29), *f* 36 (35–37) and *h* 19 (19–20). Maximum width of expanded setae: *sc2* 12 (11–13), *c1* 8 (8–9), *d* 22 (21–23), *e* 3 (3–4) and *f* 22 (21–23). All dorsal setae serrated. Bothridia *sc1* capitate with tiny spines. Setae *v1* linear; *c2* setiform; *c1* oblong very elongated; *sc2* lanceolate with three heavy dorsal veins; *d* ovate and *f* asymmetrical, both with internal cells; *e* linear, heavily serrate (Figs 28F-H); *h* elliptical, serrate with one dorsal vein. Distances between dorsal setae: *v1*–*v1* 37 (37–38), *sc2*–*sc2* 48 (47–49), *v1*–*sc2* 16 (15–18), *c1*–*c1* 45 (44–46), *c2*–*c2* 76 (74–80), *c1*–*c2* 17 (16–19), *d*–*d* 25 (23–28), *f*–*f* 11 (9–15), *e*–*f* 13 (11–16) and *h*–*h* 10 (9–14). Seta *sc2* located lateral to *sc1*. Dorsal cupules not easily seen.

Idiosoma – venter (Figs 24–25): setae *1a* 5 (4–7), inserted on tubercles posteriad of apodemes 1; *2a* 7 (6–10), posterolaterad of apodemes 2; *3a* 8 (7–11) near anteriomedial margins of apodemes 3; *3b* 6 (5–9) on posterior margins of apodemes 4. Apodeme 1 conspicuous, fused to anterior end of prosternal apodeme. Apodeme 2 short and not fused to prosternal apodeme. Prosternal apodeme not clearly united with sejugal apodeme, continuous along length to level of apodemes 2, ending in a diffuse area that reaches to sejugal apodeme. Sejugal apodeme uninterrupted. Apodeme 3 with a constriction near anterior end, extending diagonally from proximity of base of seta *3a* to anterior margin of trochanter III; apodeme 4 extending diagonally from the middle of the poststernal apodeme to base of seta *3b*. Poststernal apodeme bifurcated anteriorly. Externally, apodemes 3 and 4 surrounded by a distinct punctation. Tegula wide 16 (15–17) and very short 4 (4–5) with posterior margin slightly arched. Seta *ps* 6 (15–6) smooth.

Legs (Fig. 26): lengths (measured from femur to tarsus): leg I 44 (43–48), leg II 40 (39–41), leg III 90 (88–93), leg IV 33 (31–35). Number of setae (solenidia in parentheses) on femur, genu, tibia and tarsus, respectively: leg I: 3-4-4(2)-7(1), leg II: 3-3-4-4(1), leg III: 0+3-4-4. Claws medium-sized (not reduced) and hooked. Empodia of the legs I, II and III about the same size or slightly smaller compared to the respective basal stalks. Tarsal solenidion *ω* of tibiotarsus I 4 (4–5), stout, wider medially. Sensory cluster of tibia I incomplete, solenidion *φ1* 4, slender, capitate; famulus *k* 6 (6–7); both inserted at approximately the same level (Fig. 28A). Seta *d* of tibia I 21 (20–22), serrate. Solenidion *ω* of tarsus II proximally inserted, 4 long, stout, wider medially. Seta *d* of tibia II 18 (17–21), serrate. Femorogenu IV 19 (19–20); tibiotarsus IV 8 (7–9). Length of leg IV setae: *v*’ F 7 (7–8), *v*’ G 10 (9–11), *v*´ Ti 18 (19–23) and *tc*” 25 (23–28); setae *v*’ Ti and *tc*” smooth; *v*’ Ti falcate.

#### Adult male.

Unknown.

#### Type material.

Holotype female and 2 paratype females on *Theobroma cacao* L., 14°47'45"S; 39°10'18"W, Ilhéus, State of Bahia, Brazil, 10/IX/2012, A. C. Lofego and J.M. Rezende. Holotype and 2 paratype females are deposited in the DZSJRP.

#### Etymology.

The species name *tupi* is in honor of a Tupi people, one of the most important native indigenous tribes in Brazil which used to live in all coastal region where this mite species was found.

## Discussion

Some characters of *Daidalotarsonemus*, *Excelsotarsonemus* and other tarsonemids in general have been misunderstood or have not been clearly interpreted, certainly because of the reliance on only light microscopy technology. This becomes clear by comparing LT-SEM micrographs with the drawings of species described previously. The use of LT-SEM and other SEM techniques by acarologists is useful to truly understanding morphological details of the mites, and contributing to more accurate and reliable taxonomic and systematic studies.

The extension of the prodorsum over the gnathosoma in the genera *Daidalotarsonemus* and *Excelsotarsonemus* is a feature mentioned by Lindquist (1986) and Ochoa et al. (1995), respectively. Using the LT-SEM, it was observed the gnathosoma has the ability to protract and retract, being covered by the prodorsum and the coxisternal plates I (Figs 4, 18 and 25).This is a difficult character to discern using light microscopy, mainly because slide mounting distorts it by the flattening of the specimen between the slide and the coverslip, often pushing the gnathosoma forward. In the *Daidalotarsonemus* species studied, it was observed these mites are able to partially retract the gnathosoma under the propodosoma, leaving the distal part, including the palps exposed. The two species of *Excelsotarsonemus* are able to retract the entire gnathosoma, similar to turtles, under the propodosoma and over the apodemes 1.

Both genera studied, especially *Excelsotarsonemus*, have some dorsal setae (especially *sc2, c1*, *d*, *e* and *f*) with very broad and intricate folding patterns. It is not clear the function of these setae yet. Each one has strong veins that probably help it raise up and maintain itself perpendicular to the body. These sail-like setae might allow them to become airborne, gliding within the canopies and colonizing new trees (Ochoa and OConnor 1998). Setae *e* and *f*, because of their position and the way they lay above tergite H, seem to have different functions, perhaps related to protection, entrapping fungal spores and/or improving the aerodynamic characteristics of the mites. Some setae have even more complicated patterns, e.g. the setae *e* of *Excelsotarsonemus caravelis* (clearly asymmetric) and setae *f* of *Excelsotarsonemus tupi* (asymmetrical and with internal cells). Furthermore, setae *d* in both species apparently sits on the modified setae *e* or *f* like a lid (Fig. 21G, 21H, 28F, 28H). Tergite EF and its setae are supported by plate H, which is concave; both plates are partially covered by the posterior projection of tergite D (Fig. 28I).

It was noticed the production of cerotegument (Krantz 2009) over the body of both genera. Using LT-SEM, the cerotegument was captured extending over the body with fungi, lichens and bacteria accumulating on it. The cerotegument along with its attached material are shed at the edge of the tergites, especially on the propodosoma and tergites C and D (Figs 2C, 4C, 9C, 16C, 18C, 23C and 25C), indicating a way these mites might disseminate microorganisms and even plant pathogens. Although these mites were preserved in 70% alcohol for about eight months, the cerotegument still contained fungi and bacteria. The primary purpose of this substance appears to be water retention, but it also may allow the mite to cover itself in another layer of particles if the substance is sticky (Walter and Proctor 2013). This fact has important biological and agricultural implications. First, this substance allows them to carry debris over their body when they disperse between the canopies. Also, the cerotegument could protect the mite against harmful fungi, being a barrier between them and the soft cuticle. Lastly, by carrying fungi and bacteria, they may act as reservoirs of microorganisms (including plant pathogens) to their host plants, and spreading them throughout the forests and surrounding crops. More studies on the biology and feeding parameters of these genera are necessary to better understand their role and impact.

The discovery of three new mite species in such a small sampling area is remarkable. Although South America has five of the biodiversity hotspots biomes of the world (Myers et al. 2000), just 10 tarsonemid species have been described based on specimens found in this region (Lofego et al. 2005, Lofego and Gondim Jr. 2006, Lofego and Feres 2006, Lofego et al. 2007, Moraes et al. 2002). In addition, two species of *Daidalotarsonemus* and three of *Excelsotarsonemus* were found in very similar rainforest areas in Costa Rica (Ochoa et al. 1991; Ochoa et al. 1995, Ochoa and OConnor 1998). Most of these mite species in Costa Rica and Brazil were collected on cocoa trees. This tropical crop has broad leaves which are often covered with fungi and lichens, making it a perfect collecting trap of falling insects and mites from the surrounding tree canopy. Undoubtedly, there is much more to be learned of the species composition, biology and ecology of tarsonemid species present in this rainforest. It is also alarming to think about how much biological information is probably being lost even before it becomes known to the scientific community due to deforestation. For this reason, it is imperative to conduct more surveys to increase the knowledge of the fauna of Tarsonemidae and other mite families in forest canopies around the world.

## Supplementary Material

XML Treatment for Daidalotarsonemus oliveirai

XML Treatment for Excelsotarsonemus caravelis

XML Treatment for Excelsotarsonemus tupi
